# TREM2 protects from atherosclerosis by limiting necrotic core formation

**DOI:** 10.1038/s44161-024-00429-9

**Published:** 2024-03-12

**Authors:** Marie Piollet, Florentina Porsch, Giuseppe Rizzo, Frederieke Kapser, Dirk J. J. Schulz, Máté G. Kiss, Kai Schlepckow, Estrella Morenas-Rodriguez, Mustafa Orkun Sen, Julius Gropper, Sourish Reddy Bandi, Sarah Schäfer, Tobias Krammer, Alexander M. Leipold, Matthias Hoke, Mária Ozsvár-Kozma, Hannah Beneš, Martin Schillinger, Erich Minar, Melanie Roesch, Laura Göderle, Anastasiya Hladik, Sylvia Knapp, Marco Colonna, Rudolf Martini, Antoine-Emmanuel Saliba, Christian Haass, Alma Zernecke, Christoph J. Binder, Clément Cochain

**Affiliations:** 1 Institute of Experimental Biomedicine, University Hospital Würzburg, Würzburg, Germany; 2 Department of Laboratory Medicine, Medical University of Vienna, Vienna, Austria; 3 German Center for Neurodegenerative Diseases (DZNE) Munich, Munich, Germany; 4 Helmholtz Institute for RNA-based Infection Research (HIRI), Helmholtz-Center for Infection Research (HZI), Würzburg, Germany; 5 Institute of Molecular Infection Biology (IMIB), University of Würzburg, Würzburg, Germany; 6 Department of Internal Medicine II, Division of Angiology, Medical University of Vienna, Vienna, Austria; 7 Department of Medicine I, Laboratory of Infection Biology, Medical University of Vienna, Vienna, Austria; 8 Department of Pathology and Immunology, Washington University School of Medicine, St. Louis, MO USA; 9 Department of Neurology, Section of Developmental Neurobiology, University Hospital Würzburg, Würzburg, Germany; 10 Division of Metabolic Biochemistry, Faculty of Medicine, Biomedical Center (BMC), Ludwig-Maximilians-Universität München, Munich, Germany; 11 Munich Cluster for Systems Neurology (SyNergy), Munich, Germany

**Keywords:** Atherosclerosis, Foam cells

## Abstract

Atherosclerosis is a chronic disease of the vascular wall driven by lipid accumulation and inflammation in the intimal layer of arteries, and its main complications—myocardial infarction and stroke—are the leading cause of mortality worldwide^1,2^. Recent studies have identified triggering receptor expressed on myeloid cells 2 (TREM2), a lipid-sensing receptor regulating myeloid cell functions^3^, to be highly expressed in macrophage foam cells in experimental and human atherosclerosis^4^. However, the role of TREM2 in atherosclerosis is not fully known. Here we show that hematopoietic or global TREM2 deficiency increased, whereas TREM2 agonism decreased, necrotic core formation in early atherosclerosis. We demonstrate that TREM2 is essential for the efferocytosis capacities of macrophages and to the survival of lipid-laden macrophages, indicating a crucial role of TREM2 in maintaining the balance between foam cell death and clearance of dead cells in atherosclerotic lesions, thereby controlling plaque necrosis.

## Abstract (web summary)

Piollet, Porsch et al. report that the myeloid receptor TREM2 limits necrotic core formation in atherosclerosis and controls key atherosclerosis-related functions of macrophages, such as efferocytosis, lipid uptake and foam cell survival.

## Main

Atherosclerosis is initiated when low-density lipoproteins (LDLs) accumulate in the intima of arteries and undergo modifications (oxidation or aggregation^5^) rendering LDL pro-inflammatory, thus promoting the recruitment of monocytes, which differentiate into macrophages with various functions in the plaque. Macrophages take up modified lipoprotein particles and become foam cells, a hallmark of atherosclerotic plaques^2^, and play important roles in the clearance of cellular debris and apoptotic cells—a process called efferocytosis^2^. Death of foam cells combined with impaired efferocytosis promotes the formation of necrotic areas, a feature of unstable rupture-prone lesions^6^. Single-cell sequencing studies have revealed macrophage heterogeneity in atherosclerosis, notably identifying a subset of foamy triggering receptor expressed on myeloid cells 2 (TREM2)*-*expressing macrophages enriched for genes involved in lipid metabolism but displaying a non-inflammatory gene expression signature in murine and human plaques^4^. TREM2-expressing macrophages with similar transcriptional profiles were found in multiple diseases in mice and humans^3^, including non-alcoholic steatohepatitis (NASH), where we uncovered a protective role of TREM2-expressing macrophages^7^. In the present study, we sought to address the function of TREM2 in atherosclerosis and atherosclerosis-relevant macrophage functions.

**Fig. 1:**
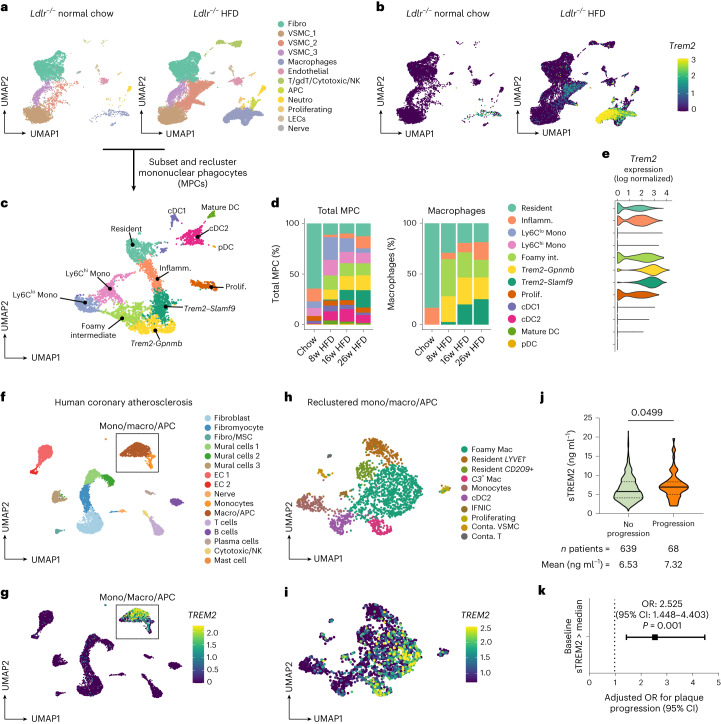
*Trem2* expression patterns at the single-cell level in mouse and human atherosclerosis. **a**, Uniform manifold approximation and projection (UMAP) visualization of scRNA-seq profiles of total mouse aortic cells in *Ldlr*^*−/−*^ mice fed normal chow or an HFD for 8 weeks, 16 weeks or 26 weeks (*Ldlr*^−/−^ HFD), *n* = 1 scRNA-seq library per timepoint. **b**, Expression of *Trem2* in murine aortas projected onto the UMAP plot, split according to experimental condition. **c**, UMAP plot of mouse aortic MPCs identified in **a** after subsetting and reclustering. Inflamm., inflammatory; Mono, monocyte; (p)DC, (plasmacytoid) dendritic cell; Prolif., proliferating. **d**, Proportion of MPC clusters among all MPCs, and macrophage clusters among all macrophages, at different times of HFD feeding. **e**, *Trem2* expression levels in MPC clusters. UMAP visualization of scRNA-seq of human atherosclerotic coronary artery cells (*n* = 4 patients, data from ref. ^9^) (**f**) and expression of *TREM2* projected onto the UMAP plot (**g**). UMAP visualization of scRNA-seq profiles of human coronary artery MPCs after subsetting and reclustering (**h**) and expression of TREM2 projected onto the UMAP plot (**i**). APC, antigen-presenting cell; Cytotoxic, cytotoxic T cell; EC, endothelial cell; Fibro, fibroblast; gdT, gammadelta T cell; LEC, lymphatic endothelial cell; MSC, mesenchymal stromal cell; Neutro, neutrophil; NK, natural killer cell. **j**, sTREM2 levels in the serum of patients with or without progression of atherosclerotic lesions from baseline to follow-up. Statistical test: unpaired two-tailed *t*-test. Center: median; dashed lines: quartiles. **k**, Plot showing adjusted OR (center of measure) and 95% CI (error bars) for progression of atherosclerotic carotid lesions from baseline to follow-up investigation after a median of 7.5 months (range, 6–9 months) in 707 patients. OR was calculated by multivariable logistic regression analysis with adjustment for age, sex, history of myocardial infarction, stroke and peripheral artery disease, arterial hypertension, smoking history, statin use, hypertension, LDL cholesterol and HbA1c. Source data

**Extended Data Fig. 1:**
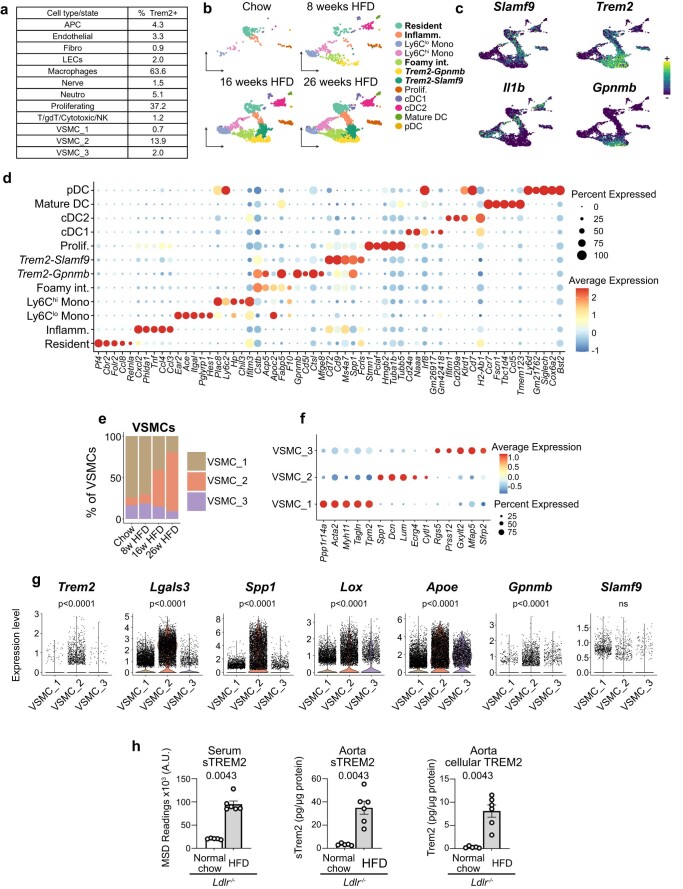
Additional scRNA-seq analysis of mouse aortic macrophages and vascular smooth muscle cells (related to Fig. 1). **a)** Percent of cells with detectable *Trem2* transcripts across aortic cell types/states in Fig. 1a; **b)** UMAP plot displayed in Fig. 1c of mononuclear phagocyte clusters split according to time of high fat diet feeding; **c)** expression of marker genes used to identify macrophage subsets projected onto the UMAP plot; **d)** dot plot showing expression of marker genes in mononuclear phagocyte populations; **e)** proportion of vascular smooth muscle cell (VSMC) clusters among total VSMCs according to time of high fat diet feeding; **f)** marker gene expression in the VSMC clusters; **g)** expression of foamy macrophage markers in the VSMC clusters. Statistical analysis performed in Seurat using ‘FindAllMarkers’ with default parameters and statistical test (Wilcoxon Rank Sum test); n cells VSMC_1: 6,618; VSMC_2: 5,143; VSCM_3: 1,945; **h)** levels of serum soluble Trem2 (sTREM2), aortic sTREM2 and aortic cellular TREM2 in *Ldlr*^*−/−*^ mice fed normal chow (n = 5) or a high fat diet (n = 6) for 6 weeks, male mice, data presented as mean +/− SEM together with individual data point distribution, statistical test: two-tailed Mann Whitney test. Source data

**Extended Data Fig. 2:**
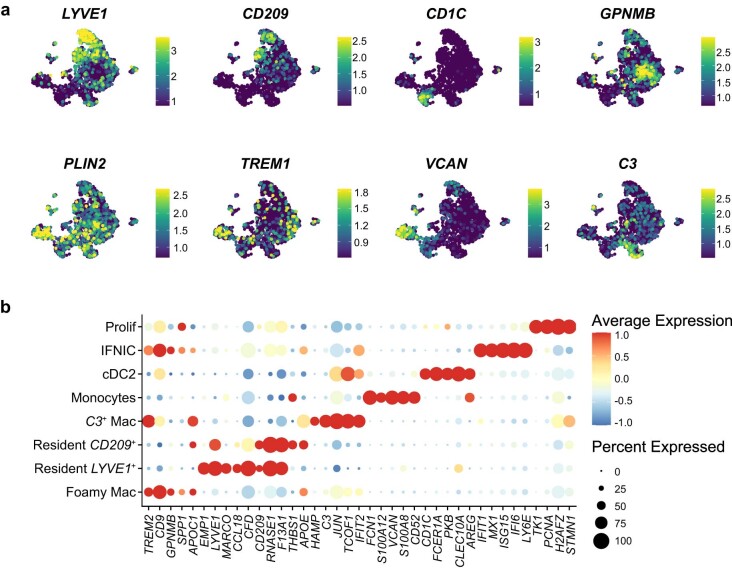
Additional scRNA-seq analysis of human atherosclerotic coronary artery mononuclear phagocytes (related to Fig. 1). **a**) Expression of the indicated transcripts projected onto the UMAP plot presented in Fig. 1i of human atherosclerotic coronary artery mononuclear phagocytes; **b**) dot plot of top marker genes used to identify human atherosclerotic coronary artery mononuclear phagocytes clusters. Prolif.: proliferating.

We analyzed *Trem2* gene expression in single-cell RNA sequencing (scRNA-seq) data of aortic cells in atherosclerotic *Ldlr*^*−/−*^ mice^8^ (Fig. 1a,b). High *Trem2* was observed in macrophages in atherosclerotic conditions (Fig. 1a,b and Extended Data Fig. 1a). In other immune lineages, few cells showed detectable *Trem2* (T cells: 1.2%, neutrophils: 5.1%) (Extended Data Fig. 1a). Analysis of mononuclear phagocytes (MPCs) revealed *Trem2* expression in various macrophage subpopulations, with the highest expression detected in lipid-associated/foamy macrophages corresponding to previously defined *Trem2*^*hi*^*Gpnmb*^*hi*^ and *Trem2*^*hi*^*Slamf9*^*hi*^ populations^4^, which accumulate during disease development (Fig. 1c–e and Extended Data Fig. 1b–d). *Trem2* expression in dendritic cells was low (Fig. 1b–e and Extended Data Fig. 1b,c). A subset of smooth muscle cells (VSMC_2) expanding at later disease stages also expressed *Trem2*, albeit at a lower level than macrophages, alongside other foamy cell markers (*Lgals3*, *Spp1* and *Apoe*) (Extended Data Fig. 1e–g). Consistent with accumulation of *Trem2*-expressing cells, we observed increased levels of total and soluble TREM2 (sTREM2) in atherosclerotic aortas and sTREM2 in the blood (Extended Data Fig. 1h). In human atherosclerotic coronary arteries^9^, *TREM2* expression was mainly restricted to MPCs but was also detected in some fibroblasts and fibromyocytes (Fig. 1f,g). *TREM2* expression was predominantly detected in foamy macrophages (Fig. 1h,i and Extended Data Fig. 2a,b). We also measured levels of sTREM2 in the plasma of 707 patients from the Inflammation and Carotid Artery–Risk for Atherosclerosis Study (ICARAS) cohort^10^. sTREM2 levels were significantly higher in patients with measurable plaque progression (Fig. 1j; *P* = 0.0499), and above-median sTREM2 levels were associated with increased risk of plaque progression (Fig. 1k; adjusted odds ratio (OR) = 2.525 (1.448–4.403); *P* = 0.001), indicating that increased sTREM2 levels are associated with plaque progression in patients.

**Fig. 2:**
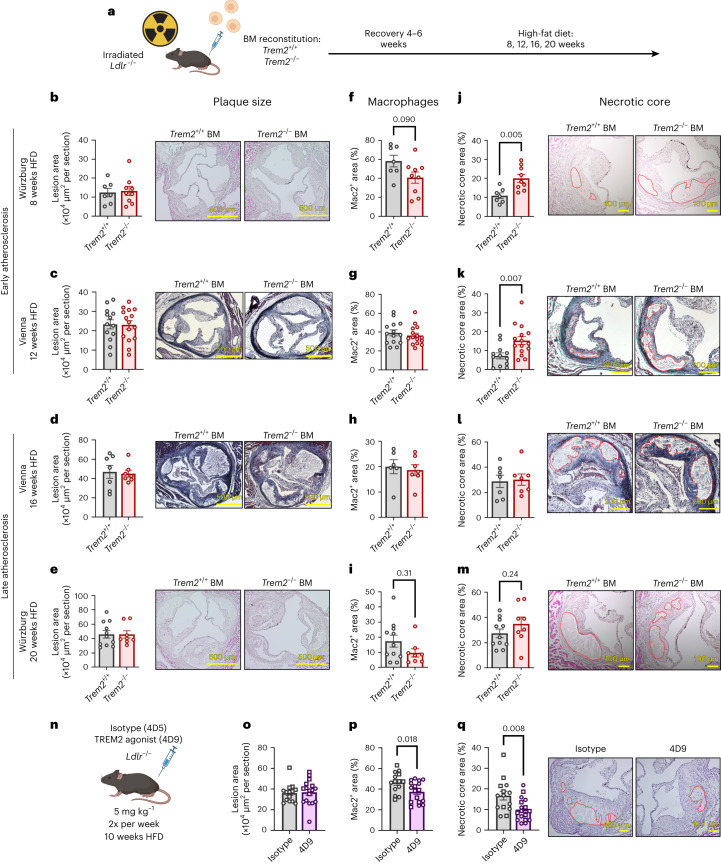
TREM2 controls necrotic core formation in early experimental atherosclerosis. **a**, Experimental design for atherogenesis experiments in BM chimeras reconstituted with *Trem2*^*+/+*^ or *Trem2*^*−/−*^ BM. Aortic sinus plaque size (**b**–**e**), macrophage content (expressed in percent of cellular plaque area) (**f**–**i**) and necrotic core size (expressed in percent of total plaque area; necrotic area is demarcated by a dashed red line) (**j**–**m**) in *Ldlr*^*−/−*^ mice irradiated and reconstituted with *Trem2*^*+/+*^ or *Trem2*^*−/−*^ BM cells and fed an HFD for 8 weeks (**b**,**f**,**j**: *n* = 7 *Trem2*^*+/+*^ BM, *n* = 9 *Trem2*^*−/−*^ BM); 12 weeks (**c**,**g**,**k**: *n* = 13 *Trem2*^*+/+*^ BM, *n* = 15 *Trem2*^*−/−*^ BM in **c**; *n* = 14 *Trem2*^*+/+*^ BM, *n* = 15 *Trem2*^*−/−*^ BM in **g**, *n* = 12 *Trem2*^*+/+*^ BM, *n* = 15 *Trem2*^*−/−*^ BM in **k**); 16 weeks (**d**,**h**,**l**: *n* = 7 *Trem2*^*+/+*^ BM, *n* = 7 *Trem2*^*−/−*^ BM in **d**,**l**; *n* = 6 *Trem2*^*+/+*^ BM, *n* = 7 *Trem2*^*−/−*^ BM in **h**); or 20 weeks (**e**,**i**,**m**: *n* = 11 *Trem2*^*+/+*^ BM, *n* = 8 *Trem2*^*−/−*^ BM). **n**, Experimental design for the in vivo TREM2 agonism experiment. Aortic sinus plaque size (**o**), macrophage content (expressed in percent of cellular plaque area) (**p**) and necrotic core size (expressed in percent of total plaque area; necrotic area is demarcated by a dashed red line) (**q**) in *Ldlr*^*−/−*^ mice fed an HFD and treated with isotype antibody or 4D9 for 10 weeks (5 mg kg^−1^ i.p. twice weekly) (*n* = 14 *Ldlr*^*−/−*^ mice treated with isotype control (eight males, six females); *n* = 17 *Ldlr*^*−/−*^ mice treated with 4D9 (10 males, seven females). Squares, female mice; circles, male mice; pooled from two experiments. All bar graphs in Fig. 2 present data as mean ± s.e.m. together with individual data point distribution. Statistical tests: two-tailed Mann–Whitney test (**b**,**d**,**f**,**h**–**j**,**l**); two-tailed unpaired *t*-test (**c**,**e**,**g**,**k**,**m**,**o**–**q**). Pictures in **a** and **n** were created with BioRender. Source data

Next, we investigated the role of TREM2 in atherosclerosis using *Ldlr*^*−/−*^ bone marrow (BM) chimeras with hematopoietic *Trem2* deficiency and fed a high-fat diet (HFD) as our main model (Fig. 2a). Experiments were performed in Würzburg (8 weeks and 20 weeks HFD) and in Vienna (12 weeks and 16 weeks HFD). Due to inter-laboratory variations, we considered these experiments as independent early (8 weeks and 12 weeks HFD) and late (16 weeks and 20 weeks HFD) lesion formation experiments rather than representing a continuum of atheroprogression. We examined aortic sinus lesions as the primary readout site.

**Extended Data Fig. 3:**
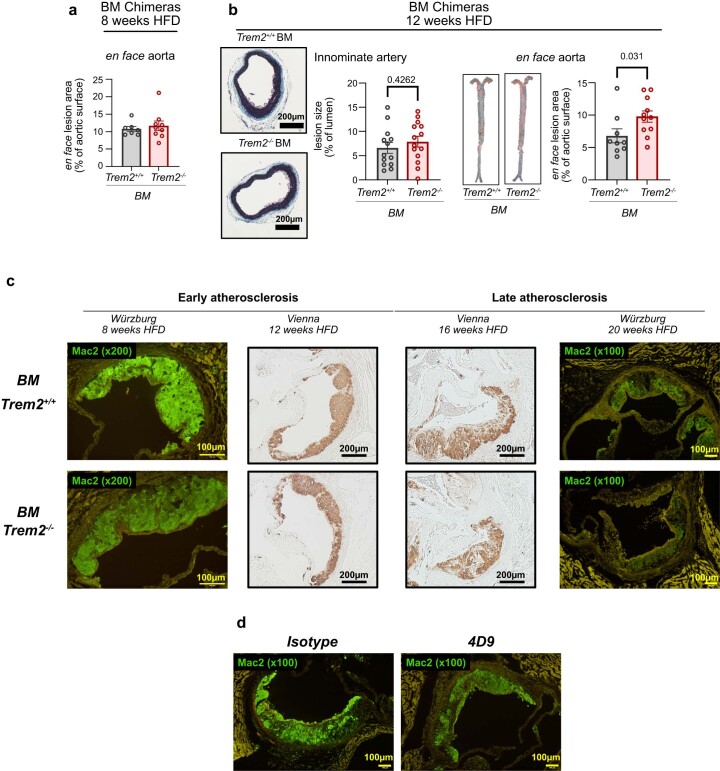
Additional analyses of atherosclerosis (related to Fig. 2). **a**) Atherosclerotic lesion formation in the aorta at 8 weeks of HFD feeding (n = 7 *Trem2*^*+/+*^ BM, n = 9 *Trem2*^*−/−*^ BM, all males) and **b**) atherosclerotic lesion formation in the innominate artery (n = 13 *Trem2*^*+/+*^ BM, n = 14 *Trem2*^*−/−*^ BM, all males) and aorta at 12 weeks of HFD (n = 9 *Trem2*^*+/+*^ BM, n = 11 *Trem2*^*−/−*^ BM, all males) in *Ldlr*^*−/−*^ mice irradiated and reconstituted with *Trem2*^*+/+*^ or *Trem2*^*−/−*^ bone marrow. Data in **a** and **b** presented as mean +/− SEM together with individual data point distribution, statistical test: two-tailed Mann Whitney test (**a**, **b** for en face aorta); unpaired two-tailed t test (**b** for innominate artery). **c**) representative pictures of macrophage coverage (Mac2 staining), related to Fig. 2f (8 weeks HFD; n = 7 *Trem2*^*+/+*^ BM, n = 9 *Trem2*^*−/−*^ BM, all males), Fig. 2g (12 weeks HFD; n = 14 *Trem2*^*+/+*^ BM, n = 15 *Trem2*^*−/−*^ BM, all males), Fig. 2h (16 weeks HFD; n = 6 *Trem2*^*+/+*^ BM, n = 7 *Trem2*^*−/−*^ BM, all males), and Fig. 2i (20 weeks HFD; n = 11 *Trem2*^*+/+*^ BM, n = 8 *Trem2*^*−/−*^ BM, all males). **d**) representative pictures of macrophage coverage (Mac2 staining) in in *Ldlr*^*−/−*^ mice fed a high fat diet and treated with isotype antibody or 4D9 for 10 weeks (5mg/kg i.p. twice weekly), related to Fig. 2p (n = 14 *Ldlr*^*−/−*^ mice treated with isotype control (8 males, 6 females); 17 *Ldlr*^*−/−*^, mice treated with 4D9 (10 males, 7 females)). Source data

**Extended Data Fig. 4:**
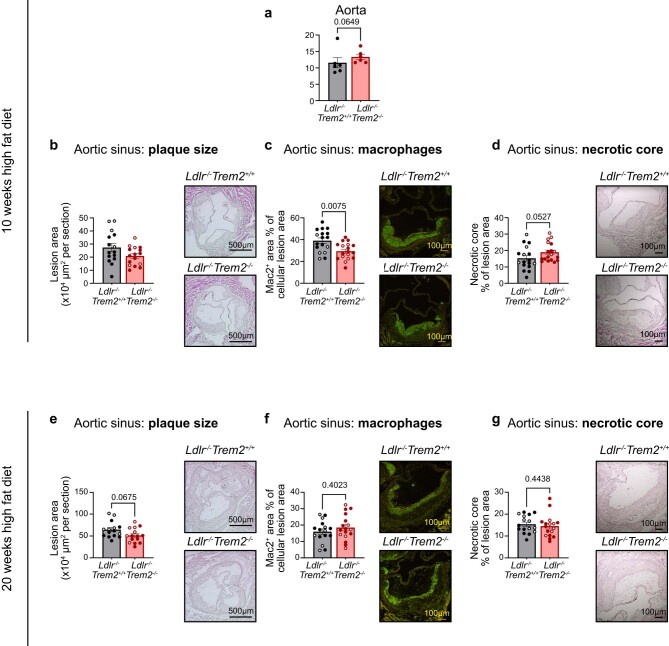
Analysis of atherosclerosis in *Ldlr*^*−/−*^*Trem2*^*−/−*^ mice. **a-d**) Analysis of atherosclerosis in *Ldlr*^*−/−*^*Trem2*^*−/−*^ mice after 10 weeks with **a**) lesion coverage in the aorta (n = 6 male mice per genotype), **b**) plaque size in the aortic sinus (*Ldlr*^*−/−*^*Trem2*^*+/+*^: 11 males, 4 females; *Ldlr*^*−/−*^*Trem2*^*−/−*^ n = 11 males, 5 females), **c**) macrophage coverage in aortic sinus lesions (expressed in percent of cellular plaque area; *Ldlr*^*−/−*^*Trem2+/+*: 12 males, 5 females; *Ldlr*^*−/−*^*Trem2*^*−/−*^ n = 12 males, 6 females) and **d**) necrotic core relative size (expressed in percent of total plaque area; *Ldlr*^*−/−*^*Trem2*^*+/+*^: 12 males, 5 females; *Ldlr*^*−/−*^*Trem2*^*−/−*^ n = 12 males, 6 females) in aortic sinus lesions; **e-g**) analysis of atherosclerosis in *Ldlr*^*−/−*^*Trem2*^*−/−*^ mice after 20 weeks of HFD feeding with **e**) plaque size in the aortic sinus (*Ldlr*^*−/−*^*Trem2*^*+/+*^: 9 males, 6 females; *Ldlr*^*−/−*^*Trem2*^*−/−*^ n = 9 males, 6 females), **f**) macrophage coverage in aortic sinus lesions (*Ldlr*^*−/−*^*Trem2*^*+/+*^: 9 males, 7 females; *Ldlr*^*−/−*^*Trem2*^*−/−*^ n = 11 males, 6 females) and **g**) necrotic core relative size in aortic sinus lesions (*Ldlr*^*−/−*^*Trem2*^*+/+*^: 9 males, 7 females; *Ldlr*^*−/−*^*Trem2*^*−/−*^ n = 11 males, 6 females). Open circles: female mice; filled circles: male mice. Data presented as mean +/− SEM together with individual data point distribution. Statistical test: two-tailed Mann Whitney test (all panels). Source data

At all timepoints, lesion size in the aortic sinus was not affected by hematopoietic *Trem2* deficiency (Fig. 2b–e). We also did not observe effects on lesion size at other sites (aorta or innominate artery) except for an increased lesion size measured in en face aortas after 12 weeks of HFD in mice with hematopoietic *Trem2* deficiency (Extended Data Fig. 3a,b). Mac2^+^ macrophage coverage within the cellular areas of lesions was not affected, except for a trend toward decrease (*P* = 0.09) at the earliest timepoint (8 weeks of HFD) in mice with *Trem2*^*−/−*^ BM (Fig. 2f–i and Extended Data Fig. 3c). During early lesion formation, we evidenced a strong increase in necrotic core size in mice with hematopoietic *Trem2* deficiency, with a 1.7-fold (8 weeks HFD; *P* = 0.005) and a more than twofold (12 weeks of HFD; *P* = 0.007) increase in independently performed experiments (Fig. 2j,k and Supplementary Fig. 1). In late-stage atherosclerosis experiments, no differences in necrotic core size were observed (Fig. 2l,m). Although we chose BM chimeras as our primary model to avoid confounding effects of TREM2 expression on non-hematopoietic cells, we also analyzed atherogenesis in *Ldlr*^*−/−*^*Trem2*^*−/−*^ mice (Extended Data Fig. 4). At 10 weeks of HFD, *Ldlr*^*−/−*^*Trem2*^*−/−*^ mice did not show any significant difference in plaque size in the aortic sinus and a trend toward increased lesion size in the aorta. Consistent with our BM chimera experiments, *Ldlr*^*−/−*^*Trem2*^*−/−*^ had decreased macrophage content and an increased necrotic core area at this timepoint (Extended Data Fig. 4a–d). At 20 weeks of HFD, *Ldlr*^*−/−*^*Trem2*^*−/−*^ mice displayed a trend toward decreased plaque size and no differences in necrotic core (Extended Data Fig. 4e–g). Overall, data obtained in *Ldlr*^*−/−*^*Trem2*^*−/−*^ mice are in line with observations in BM chimera experiments. In our BM chimera experiments and in *Ldlr*^*−/−*^*Trem2*^*−/−*^ mice, we did not observe significant effects on body weight, and inconsistent effects on systemic lipid levels, with *Trem2* deficiency being associated with no modification, slight increases or slight reductions in total cholesterol and triglycerides in serum across experiments (Extended Data Table 1). *Trem2* deficiency increased necrotic core formation in early atherosclerosis in mice with no changes in systemic lipid parameters (BM chimeras, 12 weeks of HFD, and *Ldlr*^*−/−*^*Trem2*^*−/−*^ mice, 10 weeks of HFD) but also in mice showing decreased total cholesterol and triglyceride levels (*Trem2*^*−/−*^ BM chimeras, 8 weeks of HFD), indicating that increased necrotic core formation is not driven by dyslipidemia. Altogether, these data indicate that TREM2 deficiency is associated with increased necrotic core formation during early experimental atherogenesis.

**Extended Data Fig. 5:**
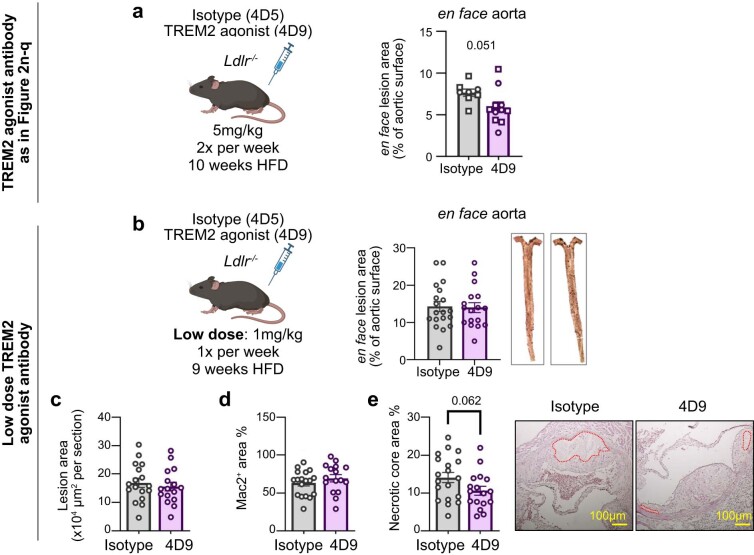
Analysis of atherosclerosis in *Ldlr*^*−/−*^ mice treated with low dose TREM2 agonist antibody 4D9. **a**) Lesion coverage in the *en face* aorta of *Ldlr*^*−/−*^ mice treated with 4D9 as in Fig. 2n-q (5mg/kg twice weekly, 10 weeks high fat diet (HFD)); n = 8 *Ldlr*^*−/−*^ mice treated with isotype control (3 males, 5 females); 11 *Ldlr*^*−/−*^ mice treated with 4D9 (5 males, 6 females), squares: female mice, circles: male mice. **b-e**) analysis of atherosclerosis in mice treated with low dose 4D9 (1mg/kg weekly, 9 weeks high fat diet) with **b**) lesion coverage in the *en face* aorta (n = 19 *Ldlr*^*−/−*^ mice treated with isotype control, n = 17 *Ldlr*^*−/−*^ mice treated with 4D9), **c**) lesion size in the aortic sinus (n = 17 *Ldlr*^*−/−*^ mice treated with isotype control, n = 16 *Ldlr*^*−/−*^ mice treated with 4D9), **d**) macrophage coverage in aortic sinus lesions (n = 18 *Ldlr*^*−/−*^ mice treated with isotype control, n = 16 *Ldlr*^*−/−*^ mice treated with 4D9) and **e**) relative necrotic core size in aortic sinus atherosclerotic lesions (n = 19 *Ldlr*^*−/−*^ mice treated with isotype control, n = 17 *Ldlr*^*−/−*^ mice treated with 4D9); data in **b-e** pooled from two experiments, all male mice. Data presented as mean +/− SEM together with individual data point distribution. Statistical test: two-tailed unpaired t test (all panels). Pictures in **a** and **b** created with BioRender.com. Source data

Next, we hypothesized that TREM2 activation might produce opposite effects—that is, reduce plaque necrosis. We treated HFD-fed *Ldlr*^*−/−*^ mice with the TREM2 agonistic antibody 4D9 (ref. ^11^) for 10 weeks, initiating treatment together with the onset of HFD (Fig. 2n). *Ldlr*^*−/−*^ mice receiving 4D9 (5 mg kg^−1^ intraperitoneally (i.p.) twice weekly) showed no differences in lesion size in the aortic sinus but a decreased macrophage content and a reduced necrotic core size (Fig. 2o–q and Extended Data Fig. 3d). Blood cholesterol or triglycerides were not affected (Extended Data Table 1). A trend toward decreased aortic plaques was observed in 4D9-treated mice (Extended Data Fig. 5a). An independent experiment employing a lower dose of 4D9 (1 mg kg^−1^ i.p. weekly) showed a clear trend toward decreased necrotic core formation, without inducing changes in lesion size or macrophage content (Extended Data Fig. 5b–e). These data demonstrate that TREM2 activation with 4D9 inhibits necrotic core formation in lesions.

Overall, our data indicate that TREM2 protects from atherosclerosis by limiting necrotic core formation. Necrotic core formation is a hallmark of unstable atherosclerotic plaques and results from a local imbalance between cell death, often associated with macrophage cholesterol overloading, and efferocytosis^6^. To gain insight into the mechanisms underlying the role of TREM2 in necrotic core formation, we performed single-nucleus RNA sequencing (snRNA-seq) and in vitro experiments.

**Fig. 3:**
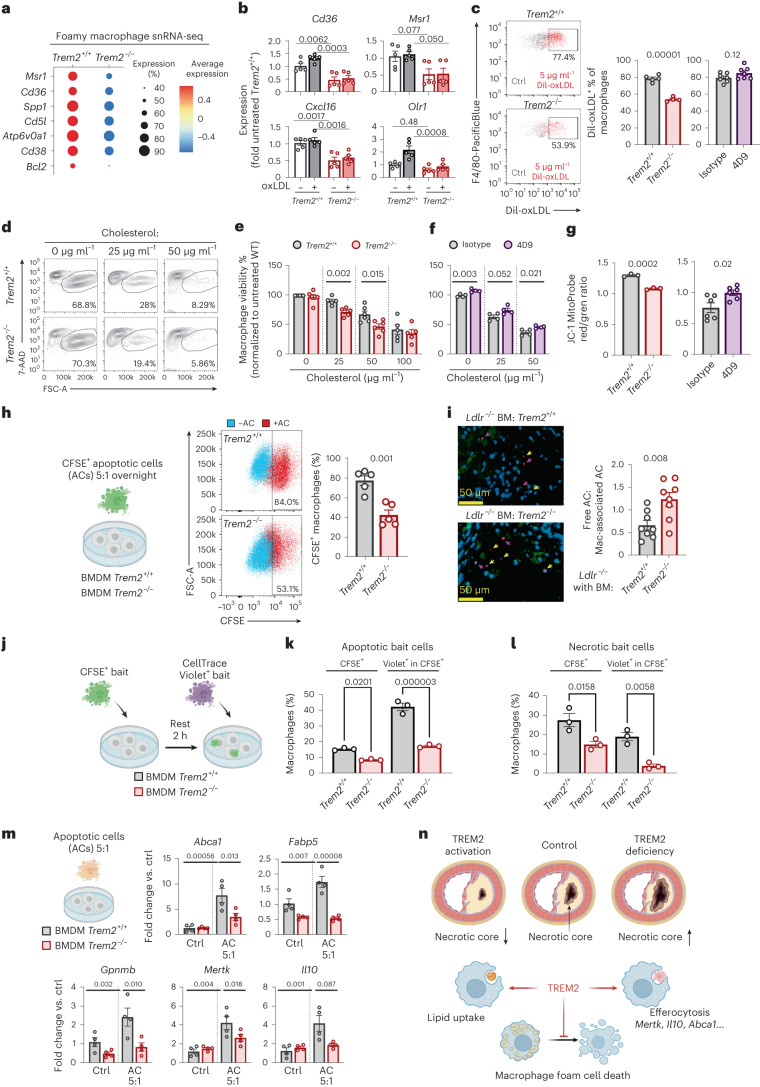
TREM2 controls macrophage survival and efferocytosis. **a**, Dot plot of differentially expressed genes in *Ldlr*^*−/−*^*Trem2*^*+/+*^ and *Ldlr*^*−/−*^*Trem2*^*−/−*^ foamy macrophages as determined by snRNA-seq analyses. **b**, Expression of the indicated transcripts in *Trem2*^*+/+*^ and *Trem2*^*−/−*^ BMDMs with or without oxLDL loading (*n* = 5 biological replicates per genotype and condition; pooled from two experiments). **c**, Dil-oxLDL uptake by *Trem2*^*+/+*^ and *Trem2*^*−/−*^ BMDMs (*n* = 5 *Trem2*^*+/+*^ and *n* = 4 *Trem2*^*−/−*^ biological replicates; representative of two independent experiments) and in BMDMs treated with the TREM2 agonistic antibody 4D9 (*n* = 7 biological replicates; pooled from two experiments). Representative flow cytometric analysis of BMDM survival in response to free cholesterol loading (**d**) and analysis of macrophage viability (expressed as percent of untreated wild-type (WT) control) (**e**) (0 µg ml^−1^ and 50 µg ml^−1^ cholesterol, *n* = 6 biological replicates per genotype; 25 µg ml^−1^ and 100 µg ml^−1^ cholesterol, *n* = 5 biological replicates per genotype; data were pooled from six experiments with BMDMs from *n* = 1 mouse from each genotype assayed in technical triplicates). **f**, Analysis of macrophage viability in response to free cholesterol loading in BMDMs treated with 4D9 or isotype control (*n* = 4 biological replicates per condition; representative of two independent experiments). **g**, JC-1 MitoProbe red/green ratio indicating mitochondrial potential in viable 7-AAD^−^ macrophages after free cholesterol loading (25 µg ml^−1^) in *Trem2*^*+/+*^ and *Trem2*^*−/−*^ BMDMs (left; *n* = 3 biological replicates per genotype and condition; one experiment) and in 4D9-treated BMDMs (right; *n* = 6 biological replicates; pooled from two experiments). **h**, Efferocytosis assay with experimental design, representative flow cytometry plots (pre-gated on viable F4/80^+^ cells) and quantitative analysis of phagocytic macrophages after overnight co-incubation with CFSE-labeled apoptotic Jurkat T cells (*n* = 5 *Trem2*^*+/+*^ and *n* = 5 *Trem2*^*−/−*^ biological replicates; pooled from two independent experiments). **i**, Analysis of in situ efferocytosis in *Ldlr*^*−/−*^ mice with hematopoietic *Trem2* deficiency after 12 weeks of HFD with representative pictures and quantification of the free apoptotic cell:macrophage-associated apoptotic cell ratio. For representative images, F4/80^+^ areas are shown in green, TUNEL^+^ areas in red and DAPI^+^ nuclei in blue. Pink arrows indicate F4/80^+^ macrophage-associated TUNEL^+^DAPI^+^ apoptotic cells, and yellow arrows indicate free TUNEL^+^DAPI^+^ apoptotic cells (*n* = 8 *Ldlr*^*−/−*^ mice with *Trem2*^*+/+*^ BM and *n* = 8 *Ldlr*^*−/−*^ mice with *Trem2*^*−/−*^ BM). **j**–**l**, Continuous efferocytosis assay with experimental design (**j**), continuous efferocytosis assay with apoptotic bait cells (**k**) and continuous efferocytosis assay with necrotic bait cells (**l**) (for **k** and **l**: *n* = 3 *Trem2*^*+/+*^ and *n* = 3 *Trem2*^*−/−*^ biological replicates; one experiment). **m**, Gene expression in *Trem2*^*+/+*^ and *Trem2*^*−/−*^ BMDMs in response to apoptotic cell efferocytosis overnight (*n* = 4 biological replicates per genotype and condition; one experiment). **n**, Proposed model and overview of the conclusions. ctrl, control; FSC-A; forward scatter area. Source data

**Extended Data Fig. 6:**
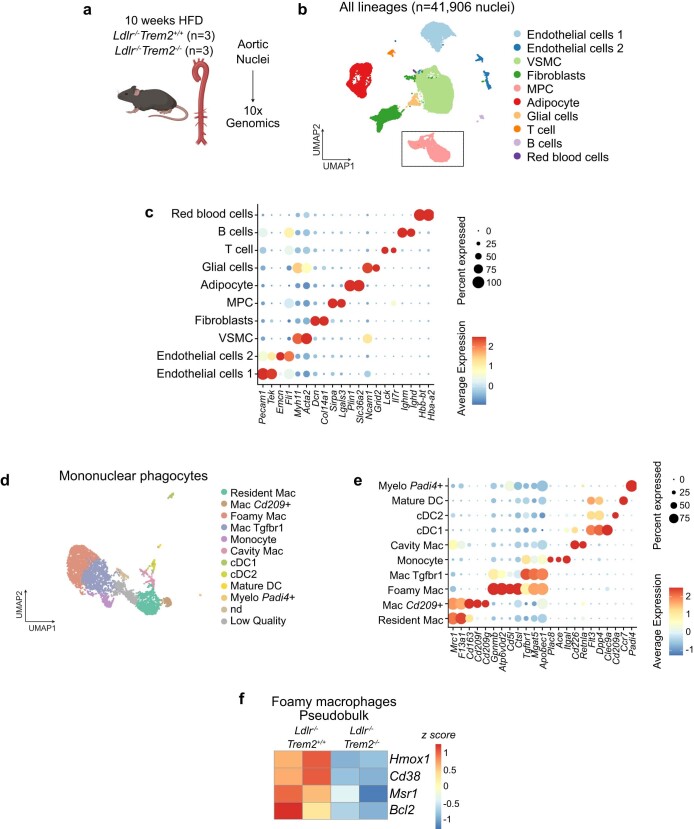
Single-nucleus RNA-seq of *Ldlr*^*−/−*^*Trem2*^*−/−*^ aortas. **a**) Experimental design; **b**) UMAP visualization of aortic single-nucleus transcriptomes from *Ldlr*^*−/−*^*Trem2*^*+/+*^ (n = 3 females) and *Ldlr*^*−/−*^*Trem2*^*−/−*^ (n = 3 females) mice after 10 weeks of HFD feeding (MPC:mononuclear phagocytes, VSMC: vascular smooth muscle cells) and **c**) expression of marker genes defining cell lineages; **d**) UMAP plot of mononuclear phagocytes after subsetting and reclustering (Mac: macrophage; Myelo: myeloid cell; DC: dendritic cell; nd: not determined); **e**) marker genes of mononuclear phagocyte clusters; **f**) expression of selected differentially expressed genes in foamy macrophages as determined from pseudo-bulk analysis of experimental replicates from the snRNA-seq data. Samples with >200 foamy macrophages were included for pseudo-bulk analysis. Picture in **a** created with BioRender.com.

snRNA-seq of aortic cells was performed in *Ldlr*^*−/−*^*Trem2*^*+/+*^ and *Ldlr*^*−/−*^*Trem2*^*−/−*^ mice after 10 weeks of HFD (Extended Data Fig. 6a–c). We identified *Gpnmb*^+^ foamy macrophages after reclustering cells corresponding to MPCs (Extended Data Fig. 6b–e). Differential expression analysis performed on all single foamy macrophages showed reduced expression of genes encoding foamy macrophage markers (*Spp1* and *Cd5l*), scavenger receptors (*Msr1* and *Cd36*), antioxidant heme oxygenase (*Hmox1*) and anti-apoptotic BCL2 (*Bcl2*) in *Trem2*^*−/−*^ foamy macrophages (Fig. 3a). A more stringent pseudo-bulk analysis (Methods) confirmed lower expression of *Hmox1*, *Msr1* and *Bcl2* in *Trem2*^*−/−*^ foamy macrophages (Extended Data Fig. 6f). This analysis suggests that TREM2 affects expression of genes associated with the foamy macrophage signature, lipid uptake and pro-survival activities in atherosclerosis-associated foamy macrophages.

**Extended Data Fig. 7:**
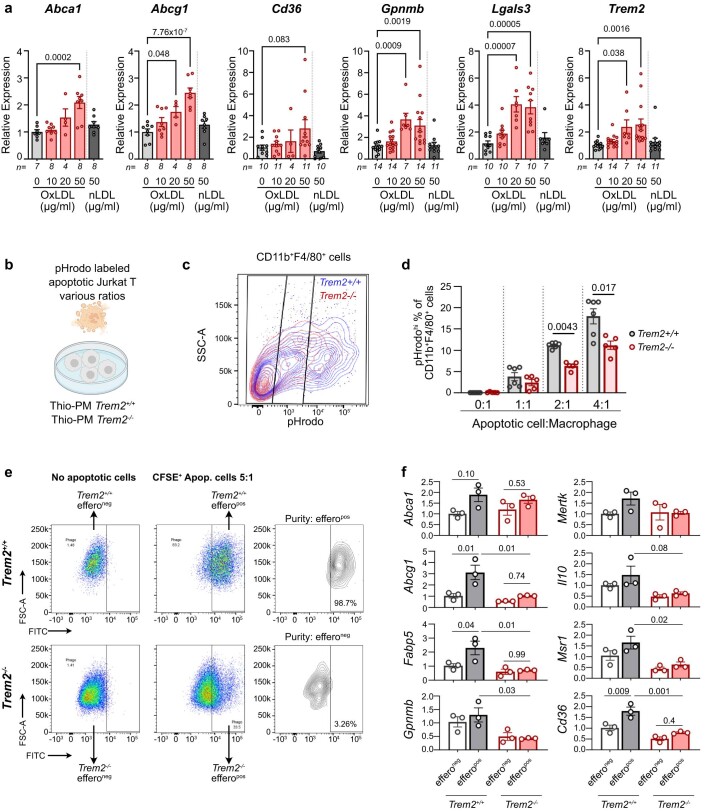
Additional in vitro assays. **a)** Gene expression in thioglycolate-elicited peritoneal macrophages (Thio-PM) in response to OxLDL and native LDL (nLDL); pooled from 2 (*Abca1*, *Abcg1*), 3 (*Cd36*, *Lgals3*) or 4 (*Gpnmb*, *Trem2*) experiments (n for each condition indicated in the figure, each data point representing a biological replicate, statistical test: 1 way ANOVA with Tukey’s test for multiple comparison); **b-d)** in vitro efferocytosis assay with Thio-PM with **b)** experimental design, **c)** representative flow cytometry plot with a 4:1 apoptotic cell (AC):macrophage ratio, and **d)** quantitative analysis (n = 6 *Trem2*^*+/+*^, n = 5 *Trem2*^*−/−*^ biological replicates; 2 pooled experiments, statistical test: two-tailed Mann Whitney test). **e)** sorting strategy to assess gene expression in efferocytic bone marrow derived macrophages (BMDM). Cells were pregated on viable F4/80^+^ macrophages. Effero^pos^: efferocytosis-positive, Effero^neg^: efferocytosis-negative; **f)** gene expression in efferocytic and non-efferocytic BMDM as sorted in **e** (gene expression normalized to *Hprt* and expressed as fold of control that is *Trem2*^*+/+*^ effero^neg^; n = 3 *Trem2*^*+/+*^, n = 3 *Trem2*^*−/−*^ biological replicates; 1 experiment, statistical test: 1 way ANOVA with Tukey’s test for multiple comparison). One biological replicate: macrophages from one mouse. All bar graphs in Extended Data Fig. 7 presented as mean +/− SEM together with individual data point distribution. Picture in **b** created with BioRender.com. Source data

In vitro, we tested whether TREM2 influences macrophage foam cell formation, survival and efferocytosis. Exposure of thioglycolate-elicited peritoneal macrophages to copper-oxidized LDL (Cu-oxLDL) induced expression of *Trem2* and other markers of the foamy/lipid-associated macrophage signature (Extended Data Fig. 7a). *Trem2*^*−/−*^ bone-marrow-derived macrophages (BMDMs) showed reduced expression of oxidized LDL (oxLDL) uptake receptors (*Cd36*, *Msr1*, *Cxcl16* and *Olr1*) at baseline and after oxLDL loading, in line with our snRNA-seq findings (Fig. 3a,b). Macrophage uptake of Dil-labeled oxLDL was reduced in *Trem2*^*−/−*^ BMDMs and showed a trend toward increase in 4D9-treated macrophages (Fig. 3c and Supplementary Fig. 2). These results indicate that TREM2 modulates oxLDL uptake by macrophages, possibly via regulation of scavenger receptor expression.

We evaluated macrophage survival in response to free cholesterol loading as an atherosclerosis-relevant in vitro model of macrophage death^12^*. Trem2*^*−/−*^ BMDMs showed reduced survival upon free cholesterol loading (Fig. 3d–e), whereas the opposite was seen in 4D9-treated BMDMs (Fig. 3f), indicating that TREM2 promotes macrophage foam cell survival. Mitochondrial potential in remaining viable macrophages was reduced in *Trem2*^*−/−*^ BMDMs and increased in 4D9-treated BMDMs (Fig. 3g), consistent with previous work linking TREM2 to phagocyte metabolic fitness during stress^13^.

**Extended Data Fig. 8:**
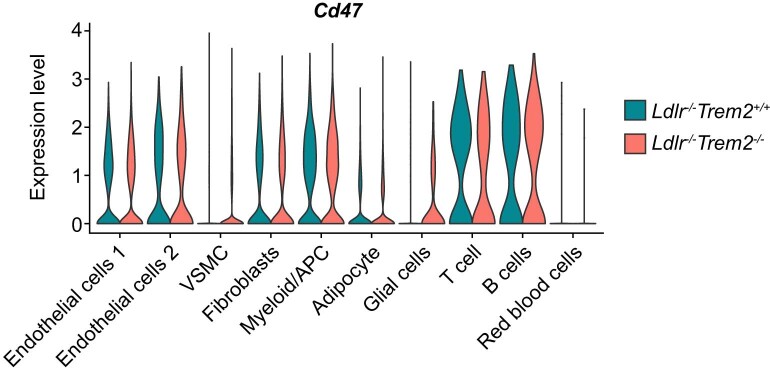
*Cd47* expression in aortic cells. Normalized *Cd47* transcript expression in single-nucleus RNA seq data in aortic cell populations identified in Supplementary Figure 6, split according to genotype.

In a flow cytometry–based assay, *Trem2*^*−/−*^ BMDMs and thioglycolate-elicited peritoneal macrophages showed reduced ability to take up apoptotic cells (Fig. 3h, Extended Data Fig. 7b–d and Supplementary Fig. 3). Analysis of in situ efferocytosis in *Ldlr*^*−/−*^ BM chimeras at 12 weeks of HFD feeding showed a higher free apoptotic cell:macrophage-associated apoptotic cell ratio in lesions of mice with *Trem2*^*−/−*^ BM, further indicating reduced efferocytic activity of macrophages within lesions (Fig. 3i). We furthermore assayed continual efferocytosis ability of BMDMs using a two-step efferocytosis assay^14^ (Fig. 3j). Primary and continuous efferocytosis of apoptotic and necrotic cells were both impaired in *Trem2*^*−/−*^ BMDMs (Fig. 3k–l), altogether suggesting an impaired ability of TREM2-deficient macrophages to clear dead cells. Efferocytosis reprograms macrophages to promote continuous efferocytosis and resolution of inflammation, notably upregulating expression of *Mertk* (ref. ^15^) and *Il10* (ref. ^16^). Although *Trem2*^*+/+*^ BMDMs upregulated *Mertk*, *Il10*, *Abca1* and markers of the foamy macrophage signature (*Gpnmb* and *Fabp5*)^4^ in response to efferocytosis, this response was blunted in *Trem2*^*−/−*^ BMDMs (Fig. 3m). To account for reduced efferocytosis ability of *Trem2*^*−/−*^ macrophages, we performed gene expression analysis of sorted efferocytic macrophages, yielding consistent results (Extended Data Fig. 7e–f). Our data suggest that TREM2-deficient macrophages have a decreased efferocytosis ability and altered gene expression responses triggered by efferocytosis. Efferocytosis in atherosclerotic lesions can be inhibited by the ‘don’t-eat-me’ signal CD47. In our snRNA-seq data, *Cd47* expression was not significantly affected in various aortic cell lineages in *Ldlr*^*−/−*^*Trem2*^*−/−*^ mice (Extended Data Fig. 8).

Based on our in vivo and in vitro observations, we propose that, by enhancing oxLDL uptake and macrophage foam cell survival and by increasing macrophage efferocytosis and anti-inflammatory gene expression in response to efferocytosis, TREM2 limits atherosclerotic plaque necrosis (Fig. 3n).

In experimental atherosclerosis, hematopoietic or global TREM2 deficiency increased, whereas treating *Ldlr*^*−/−*^ mice with the TREM2 agonistic antibody 4D9 (ref. ^11^) decreased, necrotic core formation. A recent report investigating TREM2 in macrophages in atherosclerosis proposed that TREM2 promotes lipid uptake and macrophage survival and that TREM2 deficiency in macrophages decreases lesion formation^17^, thereby showing TREM2-mediated modulation of macrophage functions consistent with our observations but contrasting outcomes of in vivo disease readouts. This report used *Cx3cr1-CreERT2-Trem2*^*flox*^ mice in which maintenance of *Trem2* knockout in macrophages required continuous tamoxifen administration over several weeks, which has been linked to confounding effects on systemic lipid metabolism and plaque formation in experimental atherosclerosis^18^. A recent preprint from the same group using another TREM2 agonistic antibody in established atherosclerosis also demonstrated reduced necrotic core formation^19^, corroborating our conclusions that TREM2 signaling reduces plaque necrosis.

Whether macrophage foam cell survival is deleterious or beneficial during atherogenesis likely depends on the disease stage^20^ and is difficult to investigate in experimental mouse models where complex lesions develop within short timeframes (weeks), compared to human lesions developing over decades^21^. In early atherosclerosis, the ability of macrophages to take up lipids and engage the appropriate mechanisms to clear them from the intima, while surviving and preserving their efferocytosis ability, appears necessary to maintain local tissue homeostasis. By promoting the survival, lipid handling and efferocytosis capacities of macrophages, TREM2 would have a beneficial role in this context. Furthermore, although local macrophage death underlies plaque regression upon reversal of dyslipidemia^22^, this requires continued efferocytosis. At later stages of atherosclerosis, other mechanisms might prevail in the regulation of macrophage accumulation, survival and efferocytosis, with macrophage proliferation becoming predominant^23^ and impairment of MERTK-dependent efferocytosis underlying plaque necrosis^24^. In patients, we observed an association between sTREM2 levels and carotid plaque progression. This is in line with our previous observation of a positive association between sTREM2 levels and advanced disease in patients with NASH^7^.

Our observations of macrophage-intrinsic functions of TREM2 as a regulator of macrophage survival and efferocytosis are consistent with previous research. TREM2 is a marker of disease-associated microglia in the brain^25^ and promotes survival of these cells in conditions of metabolic stress^13^, promotes efferocytosis in the diseased liver^26^ and regulates the expression of genes involved in lipid handling after a phagocytic challenge^27^. Another recent report also proposed that TREM2 promotes macrophage lipid uptake, consistent with our results, but showed reduced atherogenesis in *Apoe*^*−/−*^*Trem2*^*−/−*^ mice^28^. These contrasting findings might be caused by the use of *Apoe*^*−/−*^ mice, as APOE and TREM2 are functionally linked^29^, and macrophage-derived APOE controls macrophage survival, apoptotic cell clearance and necrotic core formation in atherosclerosis^20^.

Previous evidence indicates that TREM2 functions in other cell types and organs might affect atherosclerosis. In diet-induced obesity, TREM2 has been proposed to control adipose tissue remodeling^30^ and to possibly affect metabolic homeostasis systemically^31^. TREM2 has a protective role in the liver in experimental NASH^7,26^. We did not observe consistent effects of TREM2 deficiency on weight gain or systemic lipid levels under atherogenic conditions, and we observed increased necrotic core formation in mice with similar, or even decreased, systemic lipid levels, indicating that TREM2 limits plaque necrosis independently of lipid levels. However, we cannot fully exclude that TREM2 functions in other organs might influence lesion formation. Although *Trem2* expression is mostly restricted to MPCs^3^, we observed expression of *Trem2* in phenotypically modulated vascular smooth muscle cells (VSMCs). Guo et al.^28^ proposed that TREM2 promotes lipid uptake by VSMCs. Uncovering the role of TREM2 in VSMCs requires further studies using appropriate cell-type-specific models. Expression of *Trem2* mRNA on non-macrophage immune cells was low in our scRNA-seq analysis, but potential effects of TREM2 in other BM-derived cells cannot be categorically excluded. Likewise, beneficial effects of the TREM2 agonist antibody 4D9 might have been caused by activating TREM2 both in macrophages and in other TREM2-expressing cells.

In conclusion, our data using TREM2 deficiency and TREM2 agonism in experimental atherosclerosis uncovered a role of TREM2 in limiting plaque necrotic core formation, a feature of unstable lesions associated with plaque rupture^32^. TREM2 might represent an attractive therapeutic target in cardiometabolic disease, as, in addition to its proposed beneficial roles in obesity and other lipid-driven diseases, such as NASH, activating TREM2 signaling may limit plaque necrosis and promote stabilization of atherosclerotic lesions.

## Methods

This study adheres to all relevant ethical regulations. Experimental studies performed in Würzburg were conducted according to Good Scientific Practice institutional guidelines of the University of Würzburg. All animal studies performed in Würzburg conform to Directive 2010/63/EU of the European Parliament and have been approved by the appropriate local authorities (Regierung von Unterfranken, Wuerzburg, Germany, Akt.-Z. 55.2-2531.01-24/13, 55.2-DMS-2532-2-287 and 55.2-DMS-2532-2-1227). All experimental studies performed in Vienna were approved by the Animal Ethics Committee of the Medical University of Vienna and the Austrian Federal Ministry of Education, Science and Research and were performed according to Good Scientific Practice and national and international institutional guidelines (license no. BMWF 66.009/0336-V/3b/2018).

### Animal models

#### Mice

*Ldlr*^*−/−*^ mice (B6.129S7-Ldlrtm1Her/J, JAX stock no. 002207) were originally obtained from The Jackson Laboratory. *Trem2*^*−/−*^ mice were originally provided by Marco Colonna (Washington University). *Trem2*^*−/−*^ *Ldlr*^*−/−*^ mice were obtained by crossing the above mouse strains in-house. All mice were on a C57BL/6J background.

#### Würzburg experiments

Mice were bred and kept in individually ventilated cages (IVCs) with a 12-h dark/light cycle and ad libitum access to sterilized food and water under barrier-specific pathogen-free conditions. Ambient temperature was maintained between 20 °C and 24 °C and humidity between 45% and 65%. Six- to eight-week-old male or female *Ldlr*^*−/−*^ and *Ldlr*^*−/−*^*Trem2*^*−/−*^ mice were fed with an atherogenic diet (15% milk fat, 1.25% cholesterol; Altromin) for 10 weeks or 20 weeks. BM chimeras were generated by lethally irradiating 6- to 8-week-old male *Ldlr*^*−/−*^ mice (9 Gy, Faxitron, CP-160). Four hours after irradiation, the mice received 5 ×10^6^ total BM cells from male *Trem2*^+/+^ or *Trem2*^*−/−*^ donors and were left to recover for 4 weeks, with neomycin sulfate (bela-pharm, 2 g L^−1^, in drinking water) as antibiotic prophylaxis during the first week. Afterwards, mice were fed an atherogenic diet (HFD) (15% milk fat, 1.25% cholesterol; Altromin) for 8 weeks or 20 weeks. For TREM2 activation experiments, 6–10-week-old *Ldlr*^*−/−*^ mice were randomized to receive weekly i.p. injections of 1 mg kg^−1^ 4D9 antibody or the appropriate isotype control (4D5)^11^ and fed an HFD for 9 weeks (low-dose experiment) or randomized to receive i.p. injections of 5 mg kg^−1^ 4D9 antibody or the appropriate isotype control (4D5) twice weekly and fed an HFD for 10 weeks (high-dose experiment). Male mice were used in the low-dose experiment, and a mix of males and females were used in the high-dose experiment. Number and sex of mice used in each experimental group are indicated in the figure legends. Sex-disaggregated data are provided in the Source data tables. All animal studies conform to Directive 2010/63/EU of the European Parliament and have been approved by the appropriate local authorities (Regierung von Unterfranken, Wuerzburg, Germany, Akt.-Z. 55.2-2531.01-24/13, 55.2-DMS-2532-2-287 and 55.2-DMS-2532-2-1227).

#### Vienna experiments

Age-matched male mice of at least 8 weeks of age were used for all experiments, and groups were kept co-housed. Mice were bred and kept in IVCs with a 12-h dark/light cycle and ad libitum access to sterilized food and water under barrier-specific pathogen-free conditions at the Core Facility for Animal Breeding and Husbandry of the Center for Biomedical Research at the Medical University of Vienna. Ambient temperature was maintained around 22 °C (20–24 °C) and humidity around 55% (45–65%). For BM transplantation studies, 8-week-old male *Ldlr*^*−/−*^ mice were *γ*-irradiated using two doses of 6 Gy spaced 4 h apart to eliminate hematopoietic cells. The BM was subsequently reconstituted with 5 × 10^6^ BM cells isolated from the femurs and tibias of 6–8-week-old male *Trem2*^*−/−*^ donor mice or male *Trem2*^*+/+*^ littermates. Recipient mice recovered for 6 weeks after BM transplantation to allow for reconstitution of the hematopoietic compartment. To induce atherosclerosis, mice were put on a high-fat, high-cholesterol diet (HFD) containing 21% milk fat and 0.21% cholesterol (TD88137, ssniiff-Spezialdiäten GmbH) with ad libitum access to pellets for 12 weeks or 16 weeks. Number of mice used in each experimental group is indicated in the figure legends. The diet pellets were previously irradiated/autoclaved for sterility. All experimental studies were approved by the Animal Ethics Committee of the Medical University of Vienna and the Austrian Federal Ministry of Education, Science and Research and were performed according to Good Scientific Practice and national and international institutional guidelines (license no. BMWF 66.009/0336-V/3b/2018).

### Histology and immunohistochemistry

Histomorphological analyses were independently performed in each laboratory by investigators blinded to the experimental conditions, without prior consultation or knowledge of results obtained in the other laboratory.

#### Würzburg experiments

##### Aorta Oil-Red-O staining for atherosclerotic lesion quantification

Mice were killed by cervical dislocation under isoflurane anesthesia. Aortas were perfused with PBS, excised and fixed in 4% paraformaldehyde (PFA) for 24 h. After removing the adventitia layer, the aortas were washed in PBS for 5 min and dipped 10 times in 60% 2-propanol. Oil-Red-O staining was performed, incubating the aortas for 15 min in Oil-Red-O staining solution. After washing with 60% 2-propanol and PBS, the aortas were mounted, and pictures were acquired with a Leica DM 4000 B LED microscope. Lesion size was assessed measuring the red staining area using ImageJ software (Fiji).

##### Aortic root preparation and histology staining

Mice were killed by cervical dislocation under isoflurane anesthesia. The hearts were exposed, perfused with PBS, excised and fixed in 4% PFA for 24 h. Aortic root sections were made with a cryostat (Leica, CM3050 S) at 4-µm thickness. For immunofluorescence staining, antigen retrieval with the citrate method was performed. The sections were then blocked for 30 min with blocking solution containing 2% mouse serum, 2% rabbit serum, 2% horse serum, 1% BSA and 0.1% Triton X-100 to prevent unspecific staining. Then, they were incubated with rat anti-mouse MAC2 (Cedarlane, CL8942AP, M3/38, 1:600) antibodies overnight at 4 °C. After washing with PBS, sections were stained with goat anti-rat Alexa Fluor 488 (Thermo Fisher Scientific, A1106, polyclonal, 1:500). Finally, the sections were mounted using VECTASHIELD (Vector Laboratories, H1200) containing DAPI, and pictures were acquired with the Leica DM 4000 B LED microscope. MAC2 fluorescence area was measured using ImageJ software. For necrotic core measurement, hematoxylin and eosin staining was performed on aortic root sections. In brief, the sections were stained with hematoxylin (Morphisto, 10231) solution for 6 min and washed shortly in distilled water and then in running tap water for 6 min. Aortic root sections were stained with eosin (Morphisto, 10177) for 6 min and then washed shortly in distilled water. The sections were dehydrated in increased ethanol concentration and xylene as the last step. The slides were mounted with a mounting medium, and pictures were acquired with the Leica DM 4000 B LED microscope. Necrotic core area and plaque size were measured using ImageJ software.

#### Vienna experiments

##### Aorta, heart and innominate artery preparation

Mice were killed by CO_2_ overdose under isofluorane anaesthesia, and blood was drawn via the vena cava. Mice were post-mortally perfused via the heart with 20 ml of PBS using a perfusion system, followed by perfusion in 3.7% formaldehyde (Merck Millipore). Perivascular tissue was removed in situ, and hearts, brachiocephalic (innominate) arteries and aortas were isolated until the iliac bifurcation and stored in 3.7% formaldehyde for 16–24 h before transfer to PBS until further processing. The ventricular ends of the hearts were cut and removed in parallel to the atria to expose the aortic origin for subsequent aortic root cross-section preparation.

##### Aorta Sudan IV staining and lesion quantification

For en face preparations, aortas were pinned onto standard wax dissection pans submerged in PBS, and remaining adventitial tissue was removed. Aortas were washed in Sørensen’s phosphate buffer (pH 7.38) and subsequently incubated for 15 min in Sudan IV Staining Solution (0.5% Sudan IV in 1:1 acetone and 70% ethanol, filtered; Sigma-Aldrich) with periodic shaking. Aortas were subsequently incubated in 80% ethanol for 5 min, followed by washing in water. Images were acquired on a Zeiss Stemi Dissecting Microscope with Axiocam 208 Color and ZEN Blue software (Carl Zeiss). Lesion size was assessed in a double-blinded fashion by computer-assisted analysis using Photoshop Elements (Adobe) and ImageJ. Data are expressed as percent of Sudan IV^+^ area per total aortic area.

##### Aortic root cross-section and innominate artery preparation and lesion quantification

For aortic root cross-sections and innominate artery sections, hearts were dehydrated and embedded in paraffin according to standard protocols by incubation with increasing concentrations of ethanol followed by xylene and paraffin. Sequential tissue sectioning was subsequently performed on a microtome (Microm GmbH). Innominate arteries were sequentially sectioned in 5-µm-thick serial sections starting from the branching point of the innominate artery toward the aortic arch. Aortic roots were sectioned in 5-µm-thick serial sections starting from the appearance of the three aortic valve leaflets. Nine sections separated by 50 µm across a distance of 400 µm from aortic root origin were used for subsequent analysis. Sections were stained using Massonʼs trichrome staining according to the manufacturer’s instructions (Sigma-Aldrich). Images were photographed on a Zeiss AxioImager A1 using AxioCam MRC5 and ZEN 2.3 Pro software (Carl Zeiss). Lesion size in all three leaflets was assessed in a double-blinded fashion by computer-assisted image analysis using Photoshop Elements and ImageJ software. For cross-section lesion sizes, data are expressed as the average total lesion size across nine sections (0–400 µm) from the aortic origin, with total lesion size defined as the sum of lesions at the three leaflets at each location. For necrotic cores, data are expressed as percent of lesional area made up of necrotic, acellular area. Necrotic cores were assessed at 150–250 µm from aortic root origin, and data are expressed as average necrotic area per lesional area per leaflet across three sections spaced 50 µm apart. For innominate arteries, brachiocephalic arteries were isolated from the mice postmortem, fixed and embedded in paraffin and sectioned in 5-µm-thick serial sections. Five sections separated by 50 µm were used for subsequent analysis in a similar fashion as aortic root cross-sections. Innominate artery lesion size is expressed as percent stenosis (that is, percent of artery lumen covered by atherosclerotic lesions).

##### Immunohistochemistry

For the assessment of lesional macrophage content, aortic root cross-sections (190–210 µm from aortic origin) were stained with anti-Mac2 antibody (BioLegend, 125401, clone M3/38, 1:1,500). In brief, after dewaxing and rehydration, slides were subjected to antigen retrieval for 20 min at 99 °C at pH 6.0 using Citrate Antigen Retrieval Solution (Sigma-Aldrich) and PBS-T washing. Cross-sections were blocked in 10% goat serum in 1% BSA–PBS–Tween 20 for 1 h, followed by 10-min incubations with avidin and then biotin in PBS (Dako, Agilent). Sections were incubated with rat anti-mouse Mac2 IgG2a antibody (BioLegend, 125401, clone M3/38, 1:1,500 in Ab dilution buffer containing 1% BSA, 0.05% sodium azide, 0.1% cold fish skin gelatin and 0.01 M PBS, pH 7.2) or rat IgG2a isotype control (Thermo Fisher Scientific, 14-4321-81, clone eBR2a, 1:1,500) at 4 °C overnight, followed by H_2_O_2_ treatment and incubation with secondary antibody fragment biotinylated goat anti-rat IgG (H + L) (1:200 in PBS–Tween 20; Vector Laboratories, BA-9401, polyclonal). Finally, slides were incubated with Streptavidin Peroxidase Polymer (Sigma-Aldrich) for 30 min, followed by DAB Substrate Chromogen solution (Dako, Agilent). Slides were dehydrated using increasing concentrations of ethanol and xylene and mounted with Entellan before image acquisition on the Zeiss AxioImager A1 using AxioCam MRC5 and ZEN 2.3 Pro software. Quantification was performed by image-assisted analysis using ImageJ software to determine Mac2^+^ areas. Data are expressed as percent of positive areas within the cellular areas of the atherosclerotic plaques.

##### Necrotic core area measurement

Necrotic cores were defined as acellular/anuclear areas in hematoxylin and eosin (Würzburg) or in Massonʼs trichrome (Vienna)-stained tissue sections (Supplementary Fig. 1). Necrotic core area is expressed relative to plaque size.

##### In situ assessment of lesional efferocytosis

Formalin-fixed, paraffin-embedded tissue sections from aortic root cross-sections were heated and deparaffinized using xylene, followed by rehydration using decreasing concentrations of ethanol (Carl Roth). Slides were subjected to antigen retrieval for 20 min at 99 °C at pH 6.0 using Citrate Antigen Retrieval Solution (Sigma-Aldrich), followed by cooling, PBS rinsing and treatment with 0.01% Triton X-100 for 30 min. Sections were then incubated in a dark, humidified chamber with TUNEL mixture (according to the manufacturer’s instructions) using the In Situ Cell Death Detection Kit TMR-Red (Roche Diagnostics) at 37 °C for 90 min. After 3×PBS washing, sections were blocked for 30 min at room temperature using 10% donkey serum (Sigma-Aldrich) in PBS supplemented with 1% BSA, followed by overnight incubation at 4 °C with a rat IgG2a anti-mouse anti-F4/80 antibody (clone BM8, eBioscience, 14-4801-82, Thermo Fisher Scientific) diluted 1:50 in a 1% BSA, 0.3% Triton X-100, 0.01 M PBS buffer. In parallel, selected adjacent sections were incubated with a rat IgG2a isotype control (clone eBR2a, eBioscience, 14-4321-81, dilution 1:50) to assess specificity. After 3×PBS washing, sections were incubated with a polyclonal Alexa Fluor 647-conjugated donkey anti-rat F(ab′)_2_ fragment against rat IgG (H + L) (Jackson ImmunoResearch, 712-606-153, polyclonal) diluted 1:100 in PBS supplemented with 1% BSA for 100 min at room temperature. After PBS washing, sections were counterstained with DAPI (Sigma Aldrich, 1:1,000 in PBS) for 10 min at room temperature. Slides were mounted in Fluoromount Aqueous Mounting Medium (Sigma-Aldrich).

For quantification, 7–10 representative images from different regions of each of the three valve leaflets at a region between 190 μm and 210 μm from the appearance of valve leaflets at the aortic origin were stained and acquired at ×20 magnification using an Axio-Imager M2 (Carl Zeiss). Representative images were additionally acquired at ×40 magnification. The number of ‘free apoptotic cells’, defined as TUNEL^+^DAPI^+^ nuclei not associated with F4/80^+^ regions, and the number of ‘macrophage-associated apoptotic cells’, defined as TUNEL^+^DAPI^+^ nuclei that were surrounded by or in contact with neighbouring F4/80^+^ cells, were counted. The ratio of free apoptotic cells to macrophage-associated apoptotic cells was used as an indicator of efferocytosis efficiency as previously described^14^.

### Blood cholesterol and triglyceride measurements

#### Würzburg experiments

Blood was collected in serum collection tubes (Sarstedt, 41-1500-005) and kept on ice until all samples were collected. After equilibrating the sample to room temperature for 30 min, they were centrifuged at 10,000 relative centrifugal force (rcf) for 5 min. The serum was aliquoted and stored at −80 °C until further use. Total cholesterol was measured using an Amplex Red Cholesterol Assay Kit (Invitrogen, A12216) according to the manufacturer’s instructions. Fluorescence intensity was measured using a microplate reader. Triglycerides were measured using an EzymChrom Triglyceride Assay Kit (BioAssay Systems, ETGA-200) according to the manufacturer’s instructions. Optical density was measured using a microplate reader.

#### Vienna experiments

Blood was collected using 23-gauge needles postmortem after more than 4 h of fasting via the vena cava into EDTA collection tubes (Greiner Bio-One). Plasma was obtained by centrifugation at 1,000*g* for 20 min at room temperature. Plasma triglyceride and cholesterol levels were measured in an ISO-15189-accredited medical laboratory under standardized conditions on Beckman Coulter AU5400 instruments using Beckman Coulter OSR6516 Reagent at the Department of Laboratory Medicine, Medical University of Vienna.

### BMDM culture generation

Femur and tibia of two hindlimbs were used for BM isolation as described in ref. ^33^. BM cells were resuspended in RPMI supplemented with 10% FCS, 100 U ml^−1^ penicillin–streptomycin and 50 µM β-mercaptoethanol, filtered (70-µm cell strainer) and washed. Cells were resuspended in medium supplemented with 15% L929 conditioned medium; cells were counted; and 2 × 10^6^ cells per milliliter were plated in a 10-cm^2^ cell culture dish. After 7 d, macrophages were detached using Accutase (Sigma-Aldrich, A6964), washed and resuspended in 15% L929 supplemented medium, and 0.4 × 10^6^ macrophages were plated per well in a 12-well plate. Cells were rested overnight before conducting experiments. Before all in vitro assays, macrophages were incubated for 4 h in starving low-serum medium (RPMI supplemented with 1% FCS)

### Thioglycolate-elicited peritoneal macrophages

To elicit peritoneal macrophages, 12–16-week-old male mice received a single dose (50 µl g^−1^ body weight) of sterile thioglycolate (Thermo Fisher Scientific, Difco Laboratories) by i.p. injection 72 h before being killed. After mice were killed, thioglycolate-elicited macrophages were harvested by peritoneal lavage using sterile PBS + 1% BSA. Cells were subsequently plated in RPMI-1640 medium supplemented with 10% heat-inactivated FCS and allowed to adhere for 2 h, after which cells were used for subsequent experiments (oxLDL loading and efferocytosis assays).

### oxLDL uptake assay

#### Würzburg experiments

Macrophages were exposed to 5 µg ml^−1^ Dil-oxLDL (Thermo Fisher Scientific, K1612) overnight. For TREM2 activation assay, cell culture plates were coated with 4D9 or isotype control antibody at 20 µg ml^−1^ in PBS at 4 °C overnight. Plates were washed once, and macrophages were plated in complete medium. The day after, macrophages were exposed to 5 µg ml^−1^ Dil-oxLDL for 6 h. Cells were washed once with PBS and detached with Accutase. Macrophages were then collected in FACS tubes, washed with PBS supplemented with 1% FCS and incubated in Fc Block (1:50, clone 93, BioLegend, 101320) for 10 min. After blocking, macrophages were stained with F4/80 e450 (1:100, eBioscience, 48-4801-82, clone BM8) and viability dye e780 (1:1,000, Thermo Fisher Scientific, 65-0865-14) for 30 min in the dark. After washing, they were resuspended in PBS supplemented with 1% FCS and read by a FACSCelesta (BD Biosciences) and analyzed with FlowJo version 10 software. All flow cytometry gating strategies are detailed in Supplementary Fig. 2.

##### Gene expression in response to oxLDL

Cultured macrophages were incubated for 6 h with oxLDL 50 µg ml^−1^. After washing, adherent macrophages were lyzed in RA1 lysis buffer (with added β-mercaptoethanol) from a NucleoSpin RNA Extraction Kit (Macherey-Nagel).

##### Vienna experiments

Foam cell formation assays were performed as previously described^7^. In brief, thioglycolate-elicited peritoneal macrophages were treated with 10 μg ml^−1^, 20 μg ml^−1^ or 50 μg ml^−1^ Cu-oxLDL or 50 μg ml^−1^ native LDL for 24 h. Cells were subsequently rinsed in serum-free PBS and processed for RNA isolation. To confirm lipid loading, cells were stained using 2 μM BODIPY 493/503 (4,4-difluoro-1,3,5,7,8-pentamethyl-4-bora-3a,4a-diaza-s-indacene; Invitrogen, Thermo Fisher Scientific), and mean fluorescence intensity (MFI) of BODIPY-493/503 was assessed by flow cytometry as previously described^7^ (data not shown).

### Viability assay

Adherent BMDMs from either *Trem2*^*+/+*^ or *Trem2*^*−/−*^ genotype were incubated with ACAT inhibitor (Sigma-Aldrich, S9318) at 2 µg ml^−1^, together with different concentrations of soluble cholesterol (Sigma Aldrich, C4951) (25 µg ml^−1^, 50 µg ml^−1^ and 100 µg ml^−1^). For TREM2 activation assay, cell culture plates were coated with 4D9 or isotype control antibody at 20 µg ml^−1^ in PBS at 4 °C overnight. Plates were washed once, and macrophages were plated in complete medium. The day after, adherent macrophages were incubated with ACAT inhibitor (Sigma-Aldrich, S9318) at 2 µg ml^−1^ and M-CSF (5 ng ml^−1^) together with different concentrations of soluble cholesterol (Sigma Aldrich, C4951) (25 µg ml^−1^ and 50 µg ml^−1^). After 24 h, cells were washed and either directly harvested or incubated for 20 min with JC-1 MitoProbe at 1:1,000 dilution (Cell Signaling Technology, 92891), washed and then detached gently with Accutase and stained with 7-AAD (Thermo Fisher Scientific), according to the manufacturerʼs instructions. The cells were read by FACSCelesta and analyzed with FlowJo version 10 software. All flow cytometry gating strategies are detailed in Supplementary Fig. 2.

### Efferocytosis assays

#### Vienna experiments

To generate bait cells, Jurkat cells were rendered apoptotic/necrotic by exposure to UV irradiation (100 mJ cm^−2^) and kept in culture at 37 °C with 5% CO_2_ for 24 h before the efferocytosis assay. Jurkat cells were subsequently stained using pH-sensitive pHrodo Red dye (Thermo Fisher Scientific) and cultured with previously plated adherent thioglycolate-elicited peritoneal macrophages at the indicated ratios (1:1, 2:1 and 4:1). pHrodo-labeled bait cells were incubated with macrophages for 2 h, after which medium containing apoptotic bait cells was removed and remaining unbound cells were washed off using PBS. Macrophages were subsequently harvested by gentle scraping and stained (anti-CD11b-FITC, clone M1/70, BioLegend, 101206, 1:800, and anti-F4/80-PerCP/Cy5.5, clone BM8, BioLegend, 123128, 1:800) in flow cytometry buffer (cold PBS supplemented with 1% BSA) to distinguish macrophages from remaining bait cells. Macrophages were assessed for apoptotic cell uptake by flow cytometry–based measurement of pHrodo-positivity of CD11b^+^F4/80^+^ cells. All flow cytometry gating strategies are detailed in Supplementary Fig. 2.

#### Würzburg experiments: generation of apoptotic/necrotic cells

Total thymocytes from the thymus of C57BL6/J mice were rendered apoptotic by overnight incubation at 37 °C in 1 µM staurosporine in RPMI supplemented with 10% FCS. Jurkat cells were fluorescently labeled in RPMI with either CFSE (CellTrace CFSE, Thermo Fisher Scientific) or violet dye (CellTrace Violet, Thermo Fisher Scientific) according to the manufacturerʼs instructions. They were left to rest at least 4 h in complete medium before apoptosis or necrosis induction. Apoptotic Jurkat T cells were generated by exposing Jurkat T cells (10 million cells at 1.10^6^ cells per milliliter in a 10-cm cell culture dish) to UV irradiation (312 nm) for 15 min, followed by 12 h rest in complete medium. Induction of apoptosis was verified by Annexin V/7-AAD staining (Supplementary Fig. 3), and preparations with more than 60% apoptotic cells and less than 10% necrotic cells were used. Necrotic Jurkat cells were generated by incubation at 56 °C for 30 min.

#### Würzburg experiments: efferocytosis and continuous efferocytosis assays

Figure 3h: macrophages were incubated overnight with 5:1 CFSE^+^ apoptotic Jurkat T cells or left untreated. Figure 3j–l: for continuous efferocytosis assays, macrophages were incubated for 2 h with CFSE^+^ apoptotic Jurkat cells (3 Jurkat cells:1 macrophage ratio), washed three times with PBS, left to rest for 2 h and then incubated with CellTrace Violet^+^ apoptotic Jurkat cells for 2 h. Alternatively, BMDMs were incubated for 1 h with CFSE^+^ necrotic Jurkat cells, washed three times, rested for 2 h and then incubated a second time with CellTrace Violet^+^ necrotic Jurkat cells for 1 h. Macrophages were washed three times and then harvested, stained with viability dye e780 (1:1,000, Thermo Fisher Scientific, 65-0865-14) and F4/80 PE-Cy7 (BioLegend, 123114, clone BM8, 1:300) and analyzed by flow cytometry. All flow cytometry gating strategies are detailed in Supplementary Fig. 2.

#### Würzburg experiments: gene expression in response to efferocytosis

Figure 3: cultured macrophages were incubated for 16 h with apoptotic thymocytes at a 5 thymocyte:1 macrophage ratio or left untreated. After extensive washing to remove unbound thymocytes, adherent macrophages were lyzed in RA1 lysis buffer (with added β-mercaptoethanol) from the NucleoSpin RNA Extraction Kit (Macherey-Nagel, 740855.50). Extended Data Fig. 7e*:* macrophages were incubated overnight with 5:1 CFSE^+^ apoptotic Jurkat T cells or left untreated. After extensive washing, macrophages were detached using Accutase (Sigma-Aldrich, A6964), washed and labeled with F4/80 e450 (eBioscience, 48-4801-82, clone BM8, 1:100) and viability dye e780 (Thermo Fisher Scientific, 65-0865-14, 1:1,000) for 30 min. Efferocytic and non-efferocytic macrophages were sorted directly into RA1 lysis buffer (with added β-mercaptoethanol) from the NucleoSpin RNA Extraction Kit using a FACSAria III (BD Biosciences) with a 100-µm nozzle. Before sorting cells in lysis buffer, greater than 95% sort purity was verified. Total RNA was extracted using the NucleoSpin RNA Extraction Kit in accordance with the manufacturer’s instructions. Equal amounts of template RNA were used for cDNA synthesis, and RNA was reverse transcribed using random hexamer primers of a First Strand cDNA Synthesis Kit (Thermo Fisher Scientific, K1612).

### RNA isolation, cDNA generation and assessment of gene expression in response to efferocytosis or cholesterol loading

#### Vienna experiments

For RNA extraction from in vitro Cu-oxLDL-treated macrophages, cells were lysed according to the manufacturer’s instructions using QIAzol Lysis Reagent (Qiagen). To generate cDNA for subsequent quantitative real-time PCR experiments, up to 0.3 μg of RNA was subsequently reverse transcribed according to the manufacturer’s instructions using a High-Capacity cDNA Reverse Transcription Kit (Applied Biosystems, Thermo Fisher Scientific). Real-time PCR was subsequently performed on a CFX96 Real Time PCR System (Bio-Rad) using a KAPA SYBR Fast Kit (Thermo Fisher Scientific), with the primers indicated below. Gene expression was normalized to 18S. *18S* forward: AGTCCCTGCCCTTTGTACACA, reverse: CGATCCCAGGGCCTCACTA; *Trem2* forward: CTACCAGTGTCAGAGTCTCCGA, reverse: CCTCGAAACTCGATGACTCCTC; *Gpnmb* forward: GGCTACTTCAGAGCCACCATCA, reverse: CTTTGCAGGTCACAGTGAAGTCC; *Lgals3* forward: AACACGAAGCAGGACAATAACTGG, reverse: GCAGTAGGTGAGCATCGTTGAC; *Cd36* forward: GCCAAGCTATTGCGACATGA, reverse: AAAAGAATCTCAATGTCCGAGACTTT; *Abca1* forward: GGAGCCTTTGTGGAACTCTTCC, reverse: CGCTCTCTTCAGCCACTTTGAG; *Abcg1* forward: GACACCGATGTGAACCCGTTTC, reverse: GCATGATGCTGAGGAAGGTCCT.

#### Würzburg experiments

Quantitative real-time PCR was performed on triplicate samples of template cDNA with PowerUp SYBR Green Master Mix (Applied Biosystems, A25742) on an Applied Biosystems QuantStudio 6 Flex Real-Time PCR System using specific primer pairs. Quantitative measurements were determined using the ΔΔCt method, with *Hprt* as the housekeeping gene. Primer sequences were as follows: *Hprt* forward: TCCTCCTCAGACCGCTTTT, reverse: CCTGGTTCATCATCGCTAATC; *Fabp5* forward: AAGCCACGGCTTTGAGGAGT, reverse: TTCACTGTGCTCTCGGTTTTG; *Gpnmb* forward: GGGCCATGAACAGTATCCCG, reverse: CCTTCTGGCATCTGGGGAAC; *Abca1* forward: AGTGATAATCAAAGTCAAAGGGACAC, reverse: AGCAACTTGGCACTAGTAACTCTG; *Il10* forward: ATTTGAATTCCCTGGGTGAGAAG, reverse: CACAGGGGAGAAATCGATGACA; *Spp1* forward: ATCTCACCATTCGGATGAGTCT, reverse: TGTAGGGACGATTGGAGTGAAA; *Mertk* forward: CAGGGCCTTTACCAGGGAGA, reverse: TGTGTGCTGGATGTGATCTTC; *Cd36* forward: GAACCACTGCTTTCAAAAACTGG, reverse: TGCTGTTCTTTGCCACGTCA; *Cxcl16* forward: CCTTGTCTCTTGCGTTCTTCC, reverse: TCCAAAGTACCCTGCGGTATC; *Msr1* forward: TTTCCCAATTCAAAAGCTGA, reverse: CCTCCGTTCAGGAGAAGTTC; *Olr1* forward: CAAGATGAAGCCTGCGAATGA, reverse: ACCTGGCGTAATTGTGTCCAC.

### scRNA-seq analysis of public datasets

#### Mouse scRNA-seq data

Sequencing data from Pan et al.^8^ were downloaded from the Gene Expression Omnibus (GSE155513), pre-processed in Cell Ranger 6.1.2 and further analyzed in Seurat version 4 (ref. ^34^). We used data from *Ldlr*^*−/−*^ mice fed normal chow or a Western diet for 8 weeks, 16 weeks or 26 weeks (that is, the following data from Gene Expression Omnibus GSE155513: GSM4705592, GSM4705593, GSM4705594, GSM4705595, GSM4705596, GSM4705597, GSM4705598 and GSM4705599). Individual datasets were pre-processed with quality control filtering in Seurat: cells containing more than 200 detected genes and genes detected in at least three cells were included in the analysis using the ‘CreateSeuratObject’ function with ‘min.features = 200’ and ‘min.cells = 3’. Quality control filtering was further performed to remove dead/damaged cells with a high proportion of mitochondrial transcripts (>10%) and outlier cells with high unique molecular identifier (UMI) numbers. All data were log normalized using the ‘NormalizeData’ function in Seurat with default parameters. Data were pooled and batch corrected using Harmony^35^ within Seurat. In total, 2,000 highly variable genes were identified using ‘FindVariableFeatures’ (with selection.method = ‘vst’). Data were scaled using ‘ScaleData’ with default parameters; principal component analysis was performed using ‘RunPCA’ with default parameters and batch corrected using ‘RunHarmony’ with default parameters. Dimensional reduction was performed using ‘RunUMAP (reduction = ‘harmony’, dims = 1:20)’, and clustering was performed at a 0.4 resolution using ‘FindNeighbors (reduction = ‘harmony’, dims = 1:20)’, followed by ‘FindClusters (resolution = 0.2)’. Positive marker genes for each cluster were identified using ‘FindAllMarkers’. To analyze MPC subsets, cells corresponding to MPCs were extracted and reclustered using a clustering resolution of 0.5.

#### Human scRNA-seq data

Data from total cells of human atherosclerotic coronary arteries^9^ were analyzed in Seurat version 3 starting from the author-provided cell count matrix (downloaded from Gene Expression Omnibus GSE131778), as we previously described in ref. ^36^ and ref. ^37^. Cells containing fewer than 200 detected genes were excluded, and genes detected in at least three cells were included in the analysis using the ‘CreateSeuratObject’ function with ‘min.features = 200’ and ‘min.cells = 3’. Further quality control filtering was performed, and cells with more than 5% mitochondrial transcripts were excluded as well as cells with an outlier number of UMIs (nCount_RNA > 15,000). A total of 10,934 cells were analyzed. As a pre-analysis indicated a substantial patient-driven batch effect, we performed batch correction using Harmony^35^ within Seurat, considering each patient as an independent sample. Data were normalized using the ‘NormalizeData’ function in Seurat with default parameters. In total, 2,000 highly variable genes were identified using ‘FindVariableFeatures’ (with selection.method = ‘vst’). Data were scaled using ‘ScaleData’ with default parameters, and principal component analysis was performed using ‘RunPCA’ with default parameters and batch corrected using ‘RunHarmony’ with default parameters. Dimensional reduction was performed using ‘RunUMAP (reduction = ‘harmony’, dims = 1:20)’, and clustering was performed at a 0.4 resolution using ‘FindNeighbors (reduction = ‘harmony’, dims = 1:20)’, followed by ‘FindClusters (resolution = 0.4)’. Positive marker genes for each cluster were identified using ‘FindAllMarkers’. Cell type annotation was performed based on expression of known cell lineage markers and on cluster annotations in ref. ^9^. To analyze MPC subsets, cells corresponding to MPCs were extracted and reclustered using a clustering resolution of 0.4.

### snRNA-seq

#### Library preparation and sequencing

Aortas from *Ldlr*^*−/−*^*Trem2*^*+/+*^ and *Ldlr*^*−/−*^*Trem2*^*−/−*^ mice fed an HFD for 10 weeks were snap frozen in liquid nitrogen and cryoconserved at −80 °C. Nuclei from *n* = 3 mice per group were isolated using Chromium Nuclei Isolation Kits (10x Genomics) according to the manufacturer’s instructions. For counting, nuclei were labeled with DAPI and counted using a fluorescence microscope and a Neubauer counting chamber (concentration range, 1,400–2,300 nuclei per microliter). Nuclei from each sample were loaded onto separate lanes of the 10x Genomics Chromium with the aim to recover 10,000 nuclei per sample, using loading volumes recommended by the manufacturer. We employed Chromium Next GEM Single Cell 3′ Kit version 3.1 (10x Genomics). Libraries were generated according to the manufacturer’s instructions. All libraries were quantified by a Qubit 3.0 Fluorometer (Thermo Fisher Scientific), and quality was checked using a 2100 Bioanalyzer with High Sensitivity DNA Kit (Agilent). Sequencing was performed using an S1 flowcell with a NovaSeq 6000 platform (Illumina) targeting 26,500 reads per nucleus. Sequencing data were demultiplexed and mapped with Cell Ranger software version 7.0.1 (10x Genomics). Mouse mm10 (Ensembl 98) reference was used for the alignment, and counting steps with intronic reads were included.

#### Analysis

Cell Ranger outputs (filtered_feature_bc_matrix) were loaded in R (version 4.3.1), pre-processed and analyzed using Seurat version 4 (ref. ^34^) and DoubletFinder version 2.0.3 (ref. ^38^). Each sample was pre-filtered as follows: nuclei with more than 3% mitochondrial transcripts were excluded, and clustering analysis was performed in Seurat using 20 principal components and a 0.2 resolution. Doublets were excluded using DoubletFinder version 2.0.3 (ref. ^38^), inputting a predicted doublet rate of 15%. The six resulting Seurat objects (one for each sample) were pooled. As all the samples were processed simultaneously (same 10x Genomics Chromium chip, sequencing in the same flow cell) and had similar quality control characteristics (number of nuclei recovered, reads per nuclei, UMI per nuclei, etc.), the data presented in this manuscript were pooled in Seurat without applying batch correction (replicating the analysis using the batch correction tool Harmony^35^ yielded consistent results; not shown). Clustering analysis of all aortic nuclei was performed using 20 principal components and a 0.1 resolution to identify vascular cell lineages. Cells corresponding to MPCs (monocytes, macrophages and dendritic cells) were identified and separately reclustered using 20 principal components and a 0.3 clustering resolution. Cluster markers were identified using ‘FindAllMarkers’ in Seurat. For pseudo-bulk differential expression analysis of foamy macrophages, raw counts for each gene in all cells from this cluster were aggregated to create a pseudo-bulk matrix for each sample. Two samples were excluded for this analysis as they had low numbers (<200 nuclei) within the foamy macrophage cluster (*Ldlr*^*−/−*^*Trem2*^*+/+*^ sample 3; *Ldlr*^*−/−*^*Trem2*^*−/−*^ sample 1). The pseudo-bulk count matrices were analyzed using DESeq2 version 1.40.2 (ref. ^39^), and an adjusted *P* value cutoff of 0.1 was applied for identification of differentially expressed genes.

### sTREM2 measurements in patients of the ICARAS

In total, 1,268 patients were enrolled in the prospective, single-center ICARAS between March 2002 and March 2003. Study design, patient selection criteria and inclusion and exclusion criteria were published previously^10^. Patients with acute cardiovascular events (including myocardial infarction, stroke, coronary intervention and peripheral vascular surgery) in the 6 months before enrollment were excluded to reflect the chronic disease stage. Serum was collected at enrollment for biochemical analyses. Mortality and cause of death according to International Classification of Diseases revision 10 (ICD-10) criteria were determined by screening of the national death register. All patients gave written informed consent. The study was designed in accordance with the Declaration of Helsinki and approved by the ethics committee and review board of the Medical University of Vienna. Of the 1,268 patients, 203 (16%) were lost to clinical follow-up. For 358 patients (28%), no plasma measurements of sTREM2 were available. The cohort was divided into two groups based on median sTREM2 levels measured (5,950 ng ml^−1^; interquartile range, 4,295–8,348 ng ml^−1^). Demographic parameters did not differ significantly between groups at baseline. At enrollment, patients underwent duplex ultrasonography investigations of extracranial internal carotid arteries (ICAs) to establish the presence of clinically asymptomatic atherosclerotic disease (defined as presence of carotid narrowing of any degree or presence of non-stenotic carotid plaque). Patients underwent another duplex ultrasonography investigation to assess unilateral or bilateral progression of atherosclerosis in ICAs after 6–9 months (median, 7.5 months). Degree of stenosis was classified into five categories: 0% to 29% (carotid plaques), 30% to 49% (advanced plaques), 50% to 69% (moderate stenosis), 70% to 89% (high-grade stenosis), 90% to 99% (subocclusive stenosis) and 100% (occlusion). Progression of atherosclerotic disease was defined as an increase of the degree of stenosis by at least one category. Progression of stenosis in either one or both ICAs was considered indicative of progressive disease. Plasma human sTREM2 was measured as previously described^7^ according to the manufacturer’s instructions using a human sTREM2 ELISA Kit (R&D Systems). Human plasma samples were diluted 1:15 in Reagent Diluent. Multivariable logistic regression analysis was applied to assess the effect of sTREM2 on progression of carotid atherosclerosis with adjustment for potential confounders (age (continuous), sex (binary), history of myocardial infarction (binary), stroke (binary) and peripheral artery (binary) disease, arterial hypertension (binary), smoking history (categorical), statin use (binary), LDL cholesterol (continuous) and HbA1c (continuous)). Results of the logistic regression models are presented as the OR and 95% confidence interval (CI).

### sTREM2 measurements in mice

Aortas were snap frozen in liquid nitrogen, stored at −80 °C and subsequently homogenized in 150 µl DEA buffer (0.2% diethylamine, 50 mM NaCl, pH 10) supplemented with protease inhibitor (P8340, Sigma-Aldrich) at 4 °C using a sample homogenizer (Precellys Evolution, Bertin Technologies) and centrifuged for 10 min at 5,000*g* (4 °C). The supernatant was ultracentrifuged (60 min, 130,000*g*, 4 °C). Then, the supernatant from the ultracentrifugation (soluble DEA fraction) was collected, and the pH was adjusted by adding 1:10 volume of 0.5 M Tris/HCl, pH 6.8. The pellet from the first centrifugation step was resuspended in 150 μl of RIPA buffer (20 mM Tris/HCl, pH 7.5, 150 mM NaCl, 1 mM EDTA, 1 mM EGTA, 1% NP-40, 1% sodium deoxycholate, 2.5 mM sodium pyrophosphate) supplemented with protease inhibitor (P8340, Sigma-Aldrich) and homogenized at 4 °C using a sample homogenizer (Precellys Evolution, Bertin Technologies). Samples were centrifuged for 10 min at 5,000*g* (4 °C) to remove debris; the supernatant was subjected to ultracentrifugation (60 min, 130,000*g*, 4 °C); and the supernatant from the ultracentrifugation was collected (cellular RIPA fraction). TREM2 levels in the DEA fraction (sTREM2) and RIPA fraction (cellular TREM2) of aortas and in the serum were measured using a Meso Scale Discovery (MSD) ELISA assay. An MSD GOLD small spot streptavidin plate (MSD, L45SA-1) was coated with blocking buffer (3% BSA, 0.05% Tween 20 in PBS, pH 7.4) overnight at 4 °C. The plate was then incubated for 90 min at room temperature with 0.125 µg ml^−1^ biotinylated polyclonal goat anti-mouse TREM2 capture antibody (R&D Systems, BAF1729) diluted in blocking buffer. The plate was then washed twice (wash buffer: 0.05% Tween 20 in PBS, pH 7.4). Then, 50 µl of sample containing 21.67 µg of protein of the soluble DEA fraction or 33.33 µg of protein of the cellular RIPA fraction were loaded onto the MSD plate for each sample. Serum samples were diluted 1:40 in sample dilution buffer (1% BSA, 0.05% Tween 20 in PBS, pH 7.4, protease inhibitor (P8340, Sigma-Aldrich)), and 50 µl was loaded onto the MSD plate. Each sample was assayed in technical duplicate. Recombinant murine TREM2 (Hölzel Diagnostika) diluted in sample dilution buffer (serum measurements), DEA buffer (with 10% 0.5 M Tris/HCl pH 6.8 added) or RIPA buffer (aortic TREM2 measurements) was used as standard. Plates were incubated for 120 min at room temperature. The plate was washed twice with wash buffer and then incubated with 1 µg ml^−1^ rat monoclonal anti-mouse TREM2 detection antibody (clone 5F4, in-house^40^) diluted in blocking buffer for 60 min at room temperature. The plate was washed twice with wash buffer and then incubated with a SULFO-TAG-labeled goat anti-rat secondary antibody (MSD, R32AH-1, 1:1,000, polyclonal) diluted in blocking buffer for 60 min at room temperature. Before measurement, the plate was washed twice with wash buffer and twice with PBS, pH 7.4. MSD read buffer was added to the plate, and the light emission at 620 nm after electrochemical stimulation was measured using an MSD Sector Imager 2400. TREM2 concentrations in aortic samples were normalized to the total loaded protein. In all incubation steps at room temperature, the plate was shaken at 300 r.p.m.

### Statistical analysis

Statistical analyses were performed using GraphPad Prism version 10. Results are expressed as mean ± s.e.m. For two-group comparisons, normal distribution of the data was assessed by a D’Agostino–Pearson test followed by an unpaired *t*-test (normally distributed data) or a non-parametric Mann–Witney test (non-normally distributed data). Data with multiple comparisons were assessed by one-way ANOVA followed by Tukey’s test for multiple comparisons. *P* values less than 0.05 were considered statistically significant.

### Reporting summary

Further information on research design is available in the Nature Portfolio Reporting Summary linked to this article.

### Supplementary information

Supplementary Figs. 1–3.

Reporting Summary

Supplementary Code 1:scRNA-seq analysis of GSE155513: mouse aortic cells (R script).

Supplementary Code 2:scRNA-seq analysis of GSE131778: human coronary artery cells (R script).

Supplementary Code 3:snRNA-seq analysis of Ldlr^−/−^Trem2^+/+^ and Ldlr^−/−^Trem2^*−/−*^ aortas (R script).

### Source data

Source Data Fig. 1 Statistical source data.

Source Data Fig. 2 Statistical source data.

Source Data Fig. 3 Statistical source data.

Source Data Extended Data Fig. 1 Statistical source data.

Source Data Extended Data Fig. 3 Statistical source data.

Source Data Extended Data Fig. 4 Statistical source data.

Source Data Extended Data Fig. 5 Statistical source data.

Source Data Extended Data Fig. 7 Statistical source data.

Source Data Extended Data Table 1 Statistical source data.

## Figures and Tables

**Extended Data Table 1:**
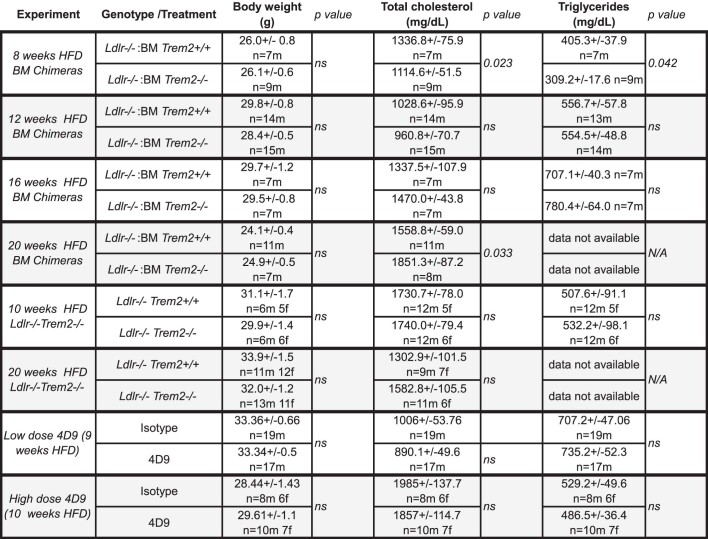
Body weight and blood lipid measurements

Body weight at sacrifice in grams and cholesterol and triglyceride levels in mg dl^−1^ in mice of all experimental groups. The number and sex of animals for each measurement is indicated directly in the table (m, male; f, female). Statistical test: two-tailed Mann–Whitney test. Data are presented as mean ± s.e.m. N/A, not applicable; NS, not significant. Isotype/4D9 low dose: 1 mg kg^−1^ weekly; Isotype/4D9 high dose: 5 mg kg^−1^ twice weekly.

Source data

## Data Availability

New sequencing data (snRNA-seq of aortic cells) have been made available from the Gene Expression Omnibus (GSE243086). Previously published scRNA-seq data from other reports and reanalyzed here are available from the Gene Expression Omnibus (mouse scRNA-seq data from ref. ^8^: GSE155513; human scRNA-seq data from ref. ^9^: GSE131778). Source data are provided with this manuscript.

## Code availability

The analysis code is provided as supplementary files.
