# Supplementary material for: TREM2 protects from atherosclerosis by limiting necrotic core formation
Source: Nat Cardiovasc Res. 2024 Mar 12;3(3):269–82. doi: 10.1038/s44161-024-00429-9 (PMC7616136; doi:10.1038/s44161-024-00429-9)
Supplement: Supplementary file 1 — Supplementary Figs. 1–3. [file 44161_2024_429_MOESM1_ESM.pdf]

nature cardiovascular research

Letter

https://doi.org/10.1038/s44161-024-00429-9

TREM2 protects from atherosclerosis by limiting necrotic core formation

In the format provided by the authors and unedited

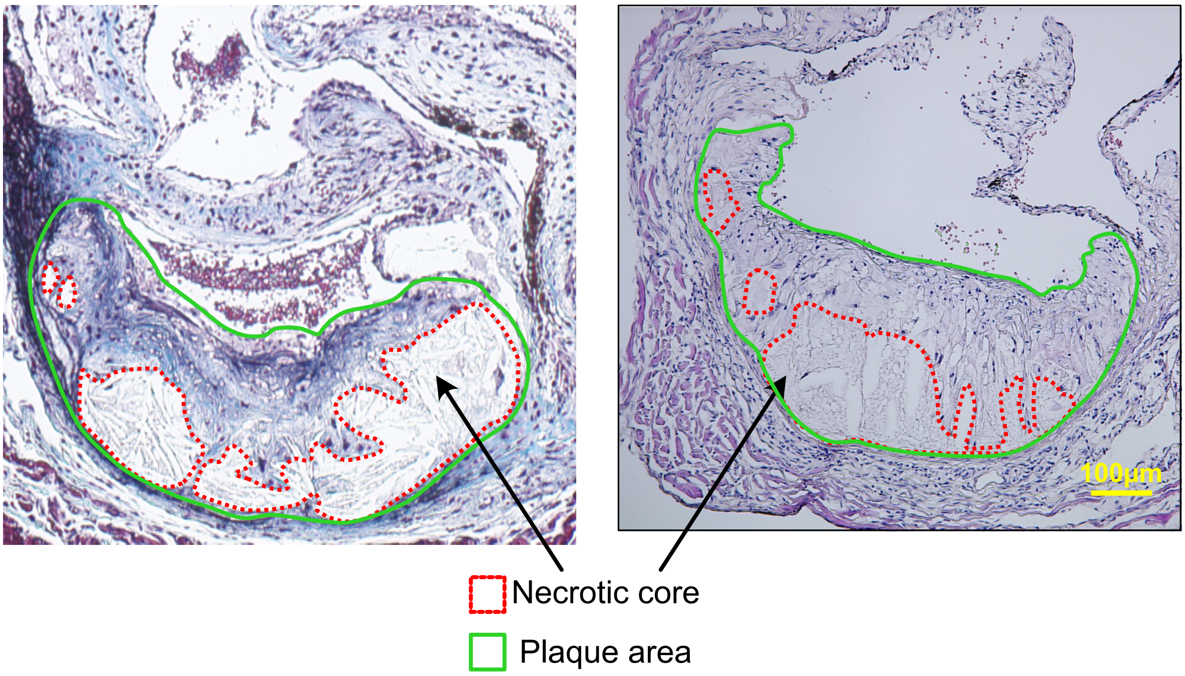

Supplementary Information Figure 1: necrotic core measurements in atherosclerotic lesions. Left: Vienna experiments, right: Würzburg experiments).

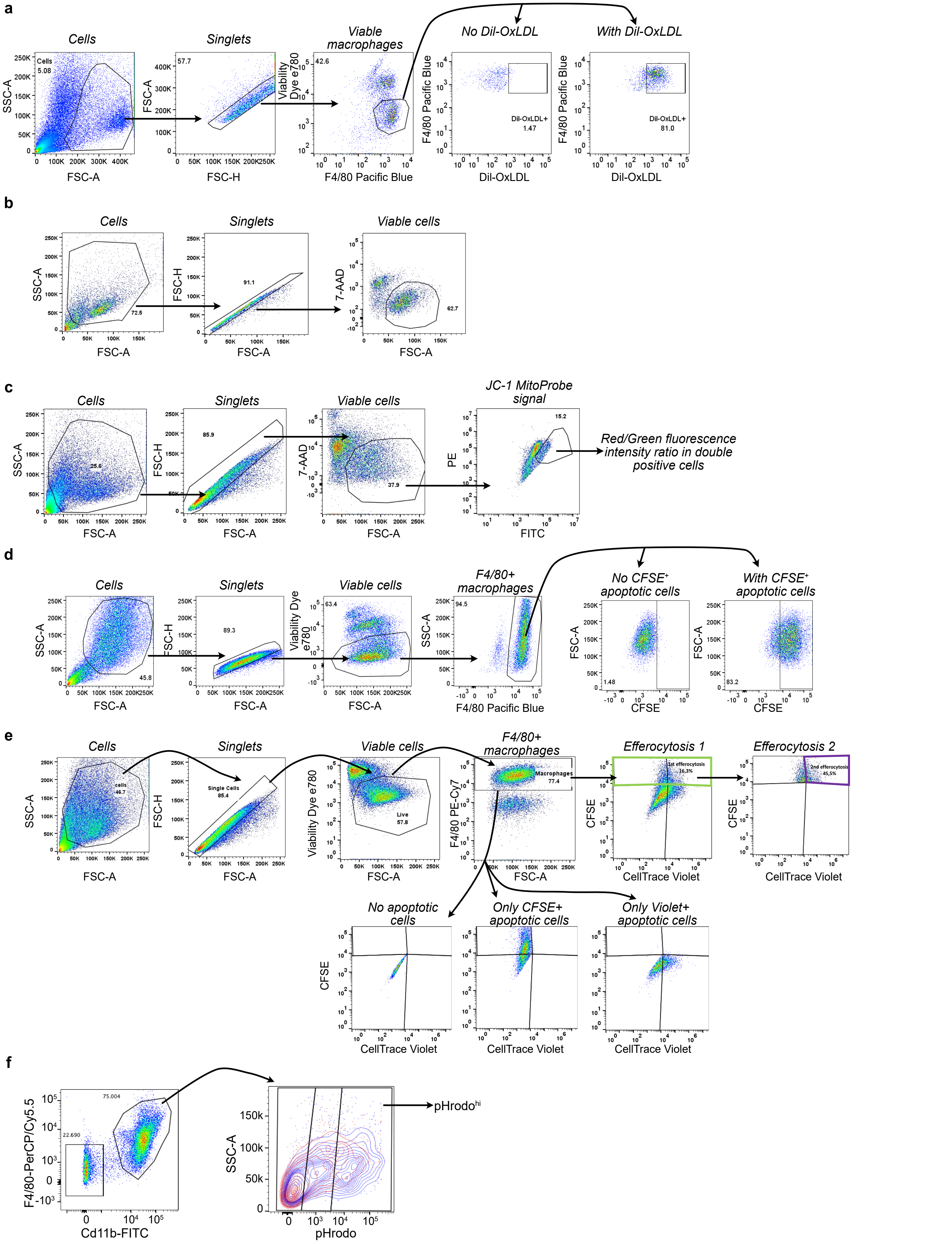

Supplementary Information Figure 2: Flow cytometry gatings. a) DilOxLDL uptake assay shown in **Figure 3c**; **b**) survival assay shown in **Figure 3d-f**; **c**) JC-1 Mitoprobe assay shown in **Figure 3g**; **d)** efferocytosis analysis and efferocytic macrophaghe sorting shown in **Figure 3h** and **Extended Data Fig. 7e**; **e**) continous efferocytosis assay shown in **Figure 3j-k**; **f**) efferocytosis assay with peritoneal macrophages shown in **Extended Data Fig. 7c**. Fixable viability dye e780 was measured in the APC-Cy7 channel; CFSE: FITC channel; CellTrace Violet: DAPI channel; Dil-OxLDL, pHrodo: PE channel.

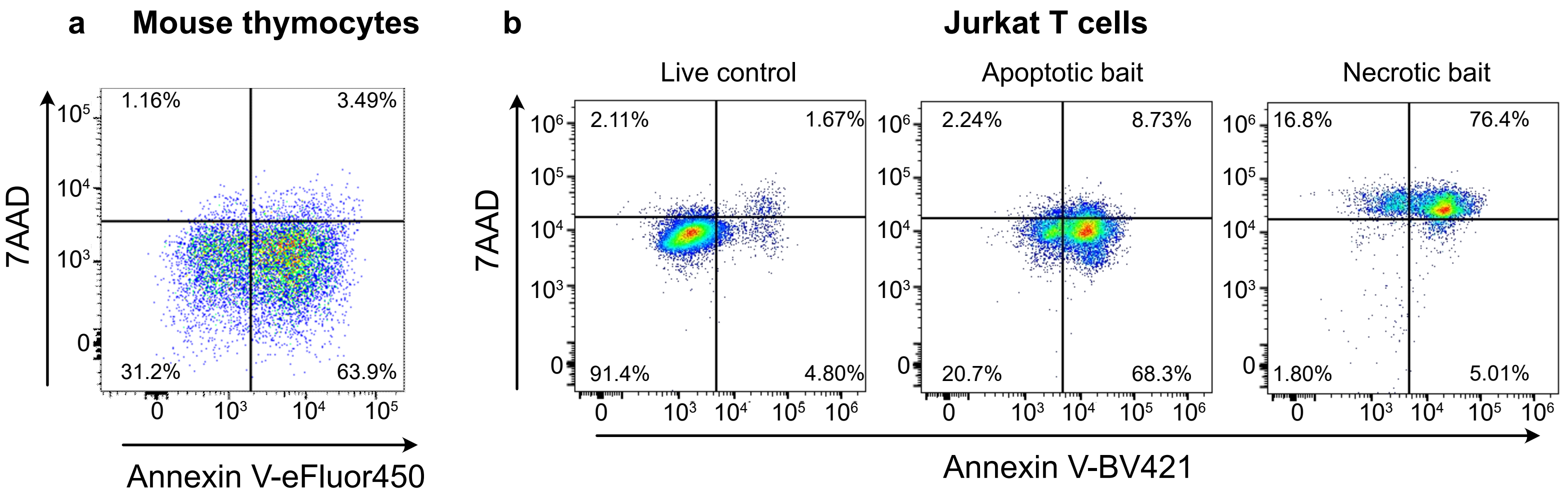

Supplementary Information Figure 3: Verification of apoptotic bait cell status for **a**) mouse thymocytes and **b**) Jurkat T cells. Necrotic cells in (**b**) were generated by heat-induced necrosis (30 minutes at 56°C)
