# Supplementary material for: TREM2 protects from atherosclerosis by limiting necrotic core formation
Source: Nat Cardiovasc Res. 2024 Mar 12;3(3):269–82. doi: 10.1038/s44161-024-00429-9 (PMC7616136; doi:10.1038/s44161-024-00429-9)
Supplement: Supplementary file 2 — Reporting Summary [file 44161_2024_429_MOESM2_ESM.pdf]

nature portfolio

Corresponding author(s): Alma Zernecke
Christoph J Binder
Clement Cochain

Last updated by author(s): Jan 11, 2024

Reporting Summary

Nature Portfolio wishes to improve the reproducibility of the work that we publish. This form provides structure for consistency and transparency in reporting. For further information on Nature Portfolio policies, see our Editorial Policies and the Editorial Policy Checklist.

Statistics

For all statistical analyses, confirm that the following items are present in the figure legend, table legend, main text, or Methods section.

n/a	Confirmed	
☐	☒	The exact sample size (*n*) for each experimental group/condition, given as a discrete number and unit of measurement
☒	☐	A statement on whether measurements were taken from distinct samples or whether the same sample was measured repeatedly
☐	☒	The statistical test(s) used AND whether they are one- or two-sided<br>*Only common tests should be described solely by name; describe more complex techniques in the Methods section.*
☒	☐	A description of all covariates tested
☐	☒	A description of any assumptions or corrections, such as tests of normality and adjustment for multiple comparisons
☐	☒	A full description of the statistical parameters including central tendency (e.g. means) or other basic estimates (e.g. regression coefficient) AND variation (e.g. standard deviation) or associated estimates of uncertainty (e.g. confidence intervals)
☐	☒	For null hypothesis testing, the test statistic (e.g. *F*, *t*, *r*) with confidence intervals, effect sizes, degrees of freedom and *P* value noted<br>*Give P values as exact values whenever suitable.*
☒	☐	For Bayesian analysis, information on the choice of priors and Markov chain Monte Carlo settings
☒	☐	For hierarchical and complex designs, identification of the appropriate level for tests and full reporting of outcomes
☒	☐	Estimates of effect sizes (e.g. Cohen's *d*, Pearson's *r*), indicating how they were calculated

Our web collection on statistics for biologists contains articles on many of the points above.

Software and code

Policy information about availability of computer code

Data collection	FACS DIVA V.8.0.1.1, Diskus Software V.5.0.6331
Data analysis	Cellranger v6.1.2; Cell Ranger v7.0.1; Seurat v3; Seurat v4 ; FlowJo v10; Graphpad Prism v10; Image J; Adobe Photoshop Elements 2020 (V18); DoubletFinder v2.0.3; Harmony v0.1.1; IBM SPSS Statistics SPSS 28.0.1.0, DESEq2 v1.40.2

For manuscripts utilizing custom algorithms or software that are central to the research but not yet described in published literature, software must be made available to editors and reviewers. We strongly encourage code deposition in a community repository (e.g. GitHub). See the Nature Portfolio guidelines for submitting code & software for further information.

Data

Policy information about availability of data

All manuscripts must include a data availability statement. This statement should provide the following information, where applicable:

- Accession codes, unique identifiers, or web links for publicly available datasets
- A description of any restrictions on data availability
- For clinical datasets or third party data, please ensure that the statement adheres to our policy

New sequencing data (snRNA-seq of aortic cells) have been made available from Gene Expression Omnibus (GSE243086). Previously published scRNA-seq data from other report and reanalyzed here are available from Gene Expression Omnibus (mouse scRNA-seq data from [8]: GSE155513; human scRNA-seq data from [9]: GSE131778). Source data are provided with this manuscript.

Research involving human participants, their data, or biological material

Policy information about studies with human participants or human data. See also policy information about sex, gender (identity/presentation), and sexual orientation and race, ethnicity and racism.

Reporting on sex and gender	see original ICARAS study PMID: 15851593
Reporting on race, ethnicity, or other socially relevant groupings	see original ICARAS study PMID: 15851593
Population characteristics	see original ICARAS study PMID: 15851593
Recruitment	see original ICARAS study PMID: 15851593
Ethics oversight	see original ICARAS study PMID: 15851593

Note that full information on the approval of the study protocol must also be provided in the manuscript.

Field-specific reporting

Please select the one below that is the best fit for your research. If you are not sure, read the appropriate sections before making your selection.

☒ Life sciences ☐ Behavioural & social sciences ☐ Ecological, evolutionary & environmental sciences

For a reference copy of the document with all sections, see nature.com/documents/nr-reporting-summary-flat.pdf

Life sciences study design

All studies must disclose on these points even when the disclosure is negative.

Sample size	Würzburg: for in vivo experiments, statistical power calculations were performed to determine sample size using G Power 3.1, and based on previous experience performing similar experiments and on commonly used sample sizes in the research field. Sc/snRNA-seq or in vitro experiments: no sample size calculations were performed; samples size were chosen based on previous experience performing similar experiments and on commonly used sample sizes in the research field. Vienna: sample sizes were based on previous experience in similar models and commonly used sample sizes in the research field.
Data exclusions	no data were excluded
Replication	Key in vivo experiments with similar experimental design were performed independently in Würzburg and Vienna labs. Trem2 agonist (4D9 antibody) treatments were performed in independent experiments using 2 different doses of antibody yielding consistent results; In vivo experiments in Ldlr-/-Trem2-/- mice were performed as complementary/confirmatory experiments to bone marrow chimera experiments; ScRNA-seq: findings were confirmed in independent datasets (Zernecke, Cardiovasc Res 2023); snRNA-seq: selected findings were validated in qPCR analysis of in vitro macrophages; qPCR gene expression experiments in response to lipid loading were performed in Vienna and Würzburg and are in part confirmatory of previously obtained data (Hendrikx, 2022); Efferocytosis: complementary experiments were performed using different time points, sources of dead cells (apoptotic/necrotic Jurkat or thymocytes), sources of macrophages (bone marrow derived macrophages, elicited peritoneal macrophages); gene expression in response to efferocytosis was assayed in two experiments with different designs yielding consistent results.
Randomization	In bone marrow chimera experiments, animals were randomized to receive either Trem2+/+ or Trem2-/- bone marrow cells. In Trem2 agonism experiments, mice were randomized to receive either 4D9 (Trem2 agonist) or 4D5 (isotype control) antibodies. In in vivo experiments comparing Ldlr-/-Trem2+/+ and Ldlr-/-Trem2-/- no randomization is possible as mice were split in groups according to genotype. In in vitro experiments using Trem2+/+ or Trem2-/- no randomization is possible as cells were split in groups according to genotype. In in vitro experiment with Trem2 agonist antibody treatment (4D9 antibody) randomization was irrelevant as the same cell preparations in standardized conditions receive either Trem2 agonist or control treatment.
Blinding	Blinding was performed where applicable i.e. for analyses of in vivo atherosclerosis readouts. Blinding was not performed for: sc/snRNA-seq analysis as these analyses utilized standardized analysis pipelines where cells from all groups are analyzed using the same parameters, qPCR gene expression analyses where all samples are analyzed using the same parameter (ct values at a predefined threshold), flow cytometry experiments where all samples were analyzed using the same gates defined using appropriate controls.

Behavioural & social sciences study design

All studies must disclose on these points even when the disclosure is negative.

Study description	*Briefly describe the study type including whether data are quantitative, qualitative, or mixed-methods (e.g. qualitative cross-sectional, quantitative experimental, mixed-methods case study).*

Research sample	*State the research sample (e.g. Harvard university undergraduates, villagers in rural India) and provide relevant demographic information (e.g. age, sex) and indicate whether the sample is representative. Provide a rationale for the study sample chosen. For studies involving existing datasets, please describe the dataset and source.*
Sampling strategy	*Describe the sampling procedure (e.g. random, snowball, stratified, convenience). Describe the statistical methods that were used to predetermine sample size OR if no sample-size calculation was performed, describe how sample sizes were chosen and provide a rationale for why these sample sizes are sufficient. For qualitative data, please indicate whether data saturation was considered, and what criteria were used to decide that no further sampling was needed.*
Data collection	*Provide details about the data collection procedure, including the instruments or devices used to record the data (e.g. pen and paper, computer, eye tracker, video or audio equipment) whether anyone was present besides the participant(s) and the researcher, and whether the researcher was blind to experimental condition and/or the study hypothesis during data collection.*
Timing	*Indicate the start and stop dates of data collection. If there is a gap between collection periods, state the dates for each sample cohort.*
Data exclusions	*If no data were excluded from the analyses, state so OR if data were excluded, provide the exact number of exclusions and the rationale behind them, indicating whether exclusion criteria were pre-established.*
Non-participation	*State how many participants dropped out/declined participation and the reason(s) given OR provide response rate OR state that no participants dropped out/declined participation.*
Randomization	*If participants were not allocated into experimental groups, state so OR describe how participants were allocated to groups, and if allocation was not random, describe how covariates were controlled.*

Ecological, evolutionary & environmental sciences study design

All studies must disclose on these points even when the disclosure is negative.

Study description	*Briefly describe the study. For quantitative data include treatment factors and interactions, design structure (e.g. factorial, nested, hierarchical), nature and number of experimental units and replicates.*
Research sample	*Describe the research sample (e.g. a group of tagged Passer domesticus, all Stenocereus thurberi within Organ Pipe Cactus National Monument), and provide a rationale for the sample choice. When relevant, describe the organism taxa, source, sex, age range and any manipulations. State what population the sample is meant to represent when applicable. For studies involving existing datasets, describe the data and its source.*
Sampling strategy	*Note the sampling procedure. Describe the statistical methods that were used to predetermine sample size OR if no sample-size calculation was performed, describe how sample sizes were chosen and provide a rationale for why these sample sizes are sufficient.*
Data collection	*Describe the data collection procedure, including who recorded the data and how.*
Timing and spatial scale	*Indicate the start and stop dates of data collection, noting the frequency and periodicity of sampling and providing a rationale for these choices. If there is a gap between collection periods, state the dates for each sample cohort. Specify the spatial scale from which the data are taken*
Data exclusions	*If no data were excluded from the analyses, state so OR if data were excluded, describe the exclusions and the rationale behind them, indicating whether exclusion criteria were pre-established.*
Reproducibility	*Describe the measures taken to verify the reproducibility of experimental findings. For each experiment, note whether any attempts to repeat the experiment failed OR state that all attempts to repeat the experiment were successful.*
Randomization	*Describe how samples/organisms/participants were allocated into groups. If allocation was not random, describe how covariates were controlled. If this is not relevant to your study, explain why.*
Blinding	*Describe the extent of blinding used during data acquisition and analysis. If blinding was not possible, describe why OR explain why blinding was not relevant to your study.*

Did the study involve field work? ☐ Yes ☐ No

Field work, collection and transport

Field conditions	*Describe the study conditions for field work, providing relevant parameters (e.g. temperature, rainfall).*
Location	*State the location of the sampling or experiment, providing relevant parameters (e.g. latitude and longitude, elevation, water depth).*
Access & import/export	*Describe the efforts you have made to access habitats and to collect and import/export your samples in a responsible manner and in*

Access & import/export	*compliance with local, national and international laws, noting any permits that were obtained (give the name of the issuing authority, the date of issue, and any identifying information).*
Disturbance	*Describe any disturbance caused by the study and how it was minimized.*

Reporting for specific materials, systems and methods

We require information from authors about some types of materials, experimental systems and methods used in many studies. Here, indicate whether each material, system or method listed is relevant to your study. If you are not sure if a list item applies to your research, read the appropriate section before selecting a response.

Materials & experimental systems

n/a	Involved in the study
☐	☒ Antibodies
☐	☒ Eukaryotic cell lines
☒	☐ Palaeontology and archaeology
☐	☒ Animals and other organisms
☒	☐ Clinical data
☒	☐ Dual use research of concern
☒	☐ Plants

Methods

n/a	Involved in the study
☒	☐ ChIP-seq
☐	☒ Flow cytometry
☒	☐ MRI-based neuroimaging

Antibodies

Antibodies used	Immunohistochemistry: anti-mouse MAC2 (Cedarlane, catalog number CL8942AP, clone M3/38, 1:600); goat anti-rat AlexaFluor488 (ThermoFisher, catalog number A1106, polyclonal, 1:500); anti-mouse mac2 IgG2a antibody (Biolegend, catalog number 125401, clone M3/38, 1:1500); rat IgG2a isotype control (Thermo Fisher, catalog number 14-4321-81, clone eBR2a, 1:1500); secondary antibody fragment biotinylated goat anti-rat IgG (H+L) (1:200 in PBS-Tween20; VectorLabs, catalog number BA-9401, polyclonal,Newark, CA, USA); rat IgG2a anti-mouse anti-F4/80 antibody (clone BM8; eBioscience, Thermo-Fisher Scientific,14-4801-82, San Diego, USA) diluted 1:50; rat igG2a isotype control (clone eBR2a, eBioscience, catalog number 14-4321-81, dilution 1:50); polyclonal Alexa Fluor 647-conjugated donkey anti-rat F(ab')2 fragment against rat IgG (H+L) (Jackson Immunoresearch, catalog number 712-606-153, polyclonal, West Grove, USA) dilution 1:100; Flow cytometry: Fc Block (1:50, Clone 93, Biolegend 101320); F4/80 e450 (1:100, eBioscience 48-4801-82, clone BM8); anti-CD16/32, unconjugated, eBioscience 14-0161-85, clone 93, 1:200; anti-CD11b-FITC (1:800; Biolegend 101206; clone M1/70); anti- F4/80-PerCP/Cy5.5 (1:800; clone BM8, Biolegend 123128); F4/80 PE-Cy7 (Biolegend 123114, clone BM8, 1:300).
Validation	Flow cytometry antibodies were validated against unlabeled samples (FMO control) or isotype-control stained samples. Immunohistochemistry antibodies were validated against isotype control stained tissue sections.

Eukaryotic cell lines

Policy information about cell lines and Sex and Gender in Research

Cell line source(s)	Jurkat T cells, originally obtained from ATCC #TIB-152 (Vienna) and DSMZ ACC 282 (Würzburg)
Authentication	no authentication was performed
Mycoplasma contamination	mycoplasma contamination was not tested
Commonly misidentified lines (See ICLAC register)	N/A

Palaeontology and Archaeology

Specimen provenance	*Provide provenance information for specimens and describe permits that were obtained for the work (including the name of the issuing authority, the date of issue, and any identifying information). Permits should encompass collection and, where applicable, export.*
Specimen deposition	*Indicate where the specimens have been deposited to permit free access by other researchers.*
Dating methods	*If new dates are provided, describe how they were obtained (e.g. collection, storage, sample pretreatment and measurement), where they were obtained (i.e. lab name), the calibration program and the protocol for quality assurance OR state that no new dates are provided.*

☐ Tick this box to confirm that the raw and calibrated dates are available in the paper or in Supplementary Information.

Ethics oversight	*Identify the organization(s) that approved or provided guidance on the study protocol, OR state that no ethical approval or guidance was required and explain why not.*

Note that full information on the approval of the study protocol must also be provided in the manuscript.

Animals and other research organisms

Policy information about studies involving animals; ARRIVE guidelines recommended for reporting animal research, and Sex and Gender in Research

Laboratory animals	Würzburg experiments: 6 to 8 week old male Ldlr-/- mice were used for bone marrow chimera experiments. Ldlr-/-Trem2-/- experiments: six to eight-week-old male or female Ldlr-/- and Ldlr-/-Trem2-/- mice were used. For Trem2 agonist antibody treatment, 6 to 8 week old Ldlr-/- mice were used. For in vitro experiments, macrophages were generated from 6 to 12 week old Trem2+/+ or Trem2-/- mice. All mice were on a C57BL6/J background. Mice were bred and kept in individually-ventilated cages (IVC) with a 12-hour dark-/light-cycle and ad libitum access to sterilized food and water under barrier-specific pathogen-free conditions (SPF). Ambient temperature was maintained between 20°C and 24°C and humidity between 45 % and 65 %. Vienna experiments: 8 week old Ldlr-/- mice were used. Mice were bred and kept in individually ventilated cages (IVC) with a 12-hour dark-/light-cycle and ad libitum access to sterilized food and water under barrier-specific pathogen-free conditions (SPF). Ambient temperature was maintained around 22°C (20-24°C) and humidity around 55% (45-65%).
Wild animals	no wild animals were used
Reporting on sex	Male and female mice were used in experimental atherosclerosis experiments. The n number of male and female mice is indicated in the respective figure legends for each experiment. Male and female mice are indicated by different symbols on graphs. Disaggregated sex data is provided in the Source Data tables.
Field-collected samples	no field-collected samples were used
Ethics oversight	Würzburg: All animal studies conform to the Directive 2010/63/EU of the European Parliament and have been approved by the appropriate local authorities (Regierung von Unterfranken, Wuerzburg, Germany, Akt.-Z. 55.2-2531.01-24/13, 55.2-DMS-2532-2-287, 55.2-DMS-2532-2-1227). Vienna: All experimental studies were approved by the Animal Ethics Committee of the Medical University of Vienna and the Austrian Federal Ministry of Education, Science and Research, and were performed according to Good Scientific Practice and national and international institutional guidelines (License number BMWF 66.009/0336-V/3b/2018).

Note that full information on the approval of the study protocol must also be provided in the manuscript.

Clinical data

Policy information about clinical studies

All manuscripts should comply with the ICMJE guidelines for publication of clinical research and a completed CONSORT checklist must be included with all submissions.

Clinical trial registration	*Provide the trial registration number from ClinicalTrials.gov or an equivalent agency.*
Study protocol	*Note where the full trial protocol can be accessed OR if not available, explain why.*
Data collection	*Describe the settings and locales of data collection, noting the time periods of recruitment and data collection.*
Outcomes	*Describe how you pre-defined primary and secondary outcome measures and how you assessed these measures.*

Dual use research of concern

Policy information about dual use research of concern

Hazards

Could the accidental, deliberate or reckless misuse of agents or technologies generated in the work, or the application of information presented in the manuscript, pose a threat to:

No	Yes	
☒	☐	Public health
☒	☐	National security
☒	☐	Crops and/or livestock
☒	☐	Ecosystems
☒	☐	Any other significant area

Experiments of concern

Does the work involve any of these experiments of concern:

No	Yes	
☒	☐	Demonstrate how to render a vaccine ineffective
☒	☐	Confer resistance to therapeutically useful antibiotics or antiviral agents
☒	☐	Enhance the virulence of a pathogen or render a nonpathogen virulent
☒	☐	Increase transmissibility of a pathogen
☒	☐	Alter the host range of a pathogen
☒	☐	Enable evasion of diagnostic/detection modalities
☒	☐	Enable the weaponization of a biological agent or toxin
☒	☐	Any other potentially harmful combination of experiments and agents

Plants

Seed stocks	*Report on the source of all seed stocks or other plant material used. If applicable, state the seed stock centre and catalogue number. If plant specimens were collected from the field, describe the collection location, date and sampling procedures.*
Novel plant genotypes	*Describe the methods by which all novel plant genotypes were produced. This includes those generated by transgenic approaches, gene editing, chemical/radiation-based mutagenesis and hybridization. For transgenic lines, describe the transformation method, the number of independent lines analyzed and the generation upon which experiments were performed. For gene-edited lines, describe the editor used, the endogenous sequence targeted for editing, the targeting guide RNA sequence (if applicable) and how the editor was applied.*
Authentication	*Describe any authentication procedures for each seed stock used or novel genotype generated. Describe any experiments used to assess the effect of a mutation and, where applicable, how potential secondary effects (e.g. second site T-DNA insertions, mosiacism, off-target gene editing) were examined.*

ChIP-seq

Data deposition

☐ Confirm that both raw and final processed data have been deposited in a public database such as GEO.

☐ Confirm that you have deposited or provided access to graph files (e.g. BED files) for the called peaks.

Data access links<br>*May remain private before publication.*	*For "Initial submission" or "Revised version" documents, provide reviewer access links. For your "Final submission" document, provide a link to the deposited data.*
Files in database submission	*Provide a list of all files available in the database submission.*
Genome browser session<br>(e.g. UCSC)	*Provide a link to an anonymized genome browser session for "Initial submission" and "Revised version" documents only, to enable peer review. Write "no longer applicable" for "Final submission" documents.*

Methodology

Replicates	*Describe the experimental replicates, specifying number, type and replicate agreement.*
Sequencing depth	*Describe the sequencing depth for each experiment, providing the total number of reads, uniquely mapped reads, length of reads and whether they were paired- or single-end.*
Antibodies	*Describe the antibodies used for the ChIP-seq experiments; as applicable, provide supplier name, catalog number, clone name, and lot number.*
Peak calling parameters	*Specify the command line program and parameters used for read mapping and peak calling, including the ChIP, control and index files used.*
Data quality	*Describe the methods used to ensure data quality in full detail, including how many peaks are at FDR 5% and above 5-fold enrichment.*
Software	*Describe the software used to collect and analyze the ChIP-seq data. For custom code that has been deposited into a community repository, provide accession details.*

Flow Cytometry

Plots

Confirm that:

☒ The axis labels state the marker and fluorochrome used (e.g. CD4-FITC).

☒ The axis scales are clearly visible. Include numbers along axes only for bottom left plot of group (a 'group' is an analysis of identical markers).

☒ All plots are contour plots with outliers or pseudocolor plots.

☒ A numerical value for number of cells or percentage (with statistics) is provided.

Methodology

Sample preparation	Adherent macrophages were washed once with PBS and detached with Accutase. Macrophages were then washed with PBS supplemented with 1% FCS, and incubated in Fc Block (1:50 in PBS+1%FCS, clone 93, Biolegend 101320) for 10 minutes. After blocking, macrophages were labeled with fluorophore conjugated antibodies for 30 minutes in the dark at 4°C. After washing in PBS+1%FCS, macrophages were resuspended in PBS+1% FCS and read using a FACS Celesta (BD) or sorted with a FACS Aria III (BD) and analyzed with Flowjo v10.
Instrument	BD FACS Celesta; BD Fortessa; BD FACS Aria III
Software	Acquisition: FACS DIVA; Analysis: FlowJo
Cell population abundance	Sorted cells were assessed for purity by flow cytometry (FACS Aria III) immediately after sorting. Data were analyzed using Flowjo v10. Sorted cells had a >95% purity.
Gating strategy	A figure detailing gating strategies is provided in the Supplementary Information. Gatings were performed as follows: Figure 3c (DilOxLDL uptake): cells (FSC-A vs. SSC-A) > Singlets (FSC-A vs. FSC-H) > viable macrophages (Viability Dye-neg F4/80+) > DilOxLDL+, negative staining gate set with a negative control (no Dil-OxLDL); Figure 3d (viability assay): cells (FSC-A vs. SSC-A) > Singlets (FSC-A vs. FSC-H) > viable cells (7-AADneg); Figure 3g (MitoProbe assay): cells (FSC-A vs. SSC-A) > Singlets (FSC-A vs. FSC-H) > viable cells (7-AADneg)> double positive red/green cells>red/green mean fluorescence intensity ratio; Figure 3h/ Extended Data Fig. 7e: cells (FSC-A vs. SSC-A) > Singlets (FSC-A vs. FSC-H) > viable cells (Viability Dye-neg) > macrophages (F4/80+) > CFSE+ vs CFSEneg, negative staining gate set with a negative control (no CFSE+ apoptotic cells). Figure 3j-k: cells (FSC-A vs. SSC-A) > Singlets (FSC-A vs. FSC-H) > viable cells (Viability Dye-neg) > macrophages (F4/80+) > CFSE+ > CellTrace Violet+, negative gate set with with a negative control (no CFSE+ apoptotic cells), a control with only CFSE+ apoptotic cells, and a control with only CellTrace Violet+ apoptotic cells. Extended Data Fig. 7c: F4/80+CD11b+ > phRodo-high, negative staining gate set with a negative control (no phRodo+ apoptotic cells).

☒ Tick this box to confirm that a figure exemplifying the gating strategy is provided in the Supplementary Information.

Magnetic resonance imaging

Experimental design

Design type	*Indicate task or resting state; event-related or block design.*
Design specifications	*Specify the number of blocks, trials or experimental units per session and/or subject, and specify the length of each trial or block (if trials are blocked) and interval between trials.*
Behavioral performance measures	*State number and/or type of variables recorded (e.g. correct button press, response time) and what statistics were used to establish that the subjects were performing the task as expected (e.g. mean, range, and/or standard deviation across subjects).*

Acquisition

Imaging type(s)	*Specify: functional, structural, diffusion, perfusion.*
Field strength	*Specify in Tesla*
Sequence & imaging parameters	*Specify the pulse sequence type (gradient echo, spin echo, etc.), imaging type (EPI, spiral, etc.), field of view, matrix size, slice thickness, orientation and TE/TR/flip angle.*
Area of acquisition	*State whether a whole brain scan was used OR define the area of acquisition, describing how the region was determined.*

Diffusion MRI ☐ Used ☐ Not used

Preprocessing

Preprocessing software	*Provide detail on software version and revision number and on specific parameters (model/functions, brain extraction, segmentation, smoothing kernel size, etc.).*
Normalization	*If data were normalized/standardized, describe the approach(es): specify linear or non-linear and define image types used for transformation OR indicate that data were not normalized and explain rationale for lack of normalization.*
Normalization template	*Describe the template used for normalization/transformation, specifying subject space or group standardized space (e.g. original Talairach, MNI305, ICBM152) OR indicate that the data were not normalized.*
Noise and artifact removal	*Describe your procedure(s) for artifact and structured noise removal, specifying motion parameters, tissue signals and physiological signals (heart rate, respiration).*
Volume censoring	*Define your software and/or method and criteria for volume censoring, and state the extent of such censoring.*

Statistical modeling & inference

Model type and settings	*Specify type (mass univariate, multivariate, RSA, predictive, etc.) and describe essential details of the model at the first and second levels (e.g. fixed, random or mixed effects; drift or auto-correlation).*
Effect(s) tested	*Define precise effect in terms of the task or stimulus conditions instead of psychological concepts and indicate whether ANOVA or factorial designs were used.*

Specify type of analysis: ☐ Whole brain ☐ ROI-based ☐ Both

Statistic type for inference (See Eklund et al. 2016)	*Specify voxel-wise or cluster-wise and report all relevant parameters for cluster-wise methods.*
Correction	*Describe the type of correction and how it is obtained for multiple comparisons (e.g. FWE, FDR, permutation or Monte Carlo).*

Models & analysis

n/a	Involved in the study
☐	☐ Functional and/or effective connectivity
☐	☐ Graph analysis
☐	☐ Multivariate modeling or predictive analysis

Functional and/or effective connectivity	*Report the measures of dependence used and the model details (e.g. Pearson correlation, partial correlation, mutual information).*
Graph analysis	*Report the dependent variable and connectivity measure, specifying weighted graph or binarized graph, subject- or group-level, and the global and/or node summaries used (e.g. clustering coefficient, efficiency, etc.).*
Multivariate modeling and predictive analysis	*Specify independent variables, features extraction and dimension reduction, model, training and evaluation metrics.*
